# Superoxide Anion Chemistry—Its Role at the Core of the Innate Immunity

**DOI:** 10.3390/ijms24031841

**Published:** 2023-01-17

**Authors:** Celia María Curieses Andrés, José Manuel Pérez de la Lastra, Celia Andrés Juan, Francisco J. Plou, Eduardo Pérez-Lebeña

**Affiliations:** 1 Hospital Clínico Universitario of Valladolid, Avenida de Ramón y Cajal, 3, 47003 Valladolid, Spain; 2 Institute of Natural Products and Agrobiology, CSIC—Spanish Research Council, Avda. Astrofísico Fco. Sánchez, 3, 38206 La Laguna, Spain; 3 Cinquima Institute and Department of Organic Chemistry, Faculty of Sciences, Valladolid University, Paseo de Belén, 7, 47011 Valladolid, Spain; 4 Institute of Catalysis and Petrochemistry, CSIC—Spanish Research Council, 28049 Madrid, Spain; 5 Sistemas de Biotecnología y Recursos Naturales, 47625 Valladolid, Spain

**Keywords:** reactive species, ROS, reactive stress, superoxide anion, innate immunity

## Abstract

Classically, superoxide anion O_2_^•−^ and reactive oxygen species ROS play a dual role. At the physiological balance level, they are a by-product of O_2_ reduction, necessary for cell signalling, and at the pathological level they are considered harmful, as they can induce disease and apoptosis, necrosis, ferroptosis, pyroptosis and autophagic cell death. This revision focuses on understanding the main characteristics of the superoxide O_2_^•−^, its generation pathways, the biomolecules it oxidizes and how it may contribute to their modification and toxicity. The role of superoxide dismutase, the enzyme responsible for the removal of most of the superoxide produced in living organisms, is studied. At the same time, the toxicity induced by superoxide and derived radicals is beneficial in the oxidative death of microbial pathogens, which are subsequently engulfed by specialized immune cells, such as neutrophils or macrophages, during the activation of innate immunity. Ultimately, this review describes in some depth the chemistry related to O_2_^•−^ and how it is harnessed by the innate immune system to produce lysis of microbial agents.

## 1. Introduction

In medicine, a great interest in the study of cellular stress and free radicals has emerged in recent years, focused on deepening our knowledge of the mechanisms of cellular self-control that allow us to improve the quality of human life and understand the origin of a large number of diseases [1].

Oxidative stress is a component of many diseases, including atherosclerosis, chronic obstructive pulmonary disease, Alzheimer’s disease and cancer, among others [2]. Simultaneously, ROS are essential for a variety of biological functions, such as cell survival, growth, proliferation and differentiation, and immune response. However, one of the major obstacles to understanding the role of these species is the lack of adequate methods to detect ROS/RNS in vivo, mainly due to their very short lifetimes and the presence of several antioxidants in cells [3]. In fact, radicals are continuously generated by most organisms as a result of the use of O_2_ as a terminal electron acceptor in the mitochondrial electron transport chains and in cytochrome P450 [4].

The term reactive species refers to two types of molecules: free radicals and non-radicals [5]. This set of molecules is formed as a result of cellular metabolism and is represented in biological systems by reactive oxygen species ROS and reactive nitrogen species RNS, which arise in both normal physiological and pathological processes. Not excluding that, there are also reactive species from other elements, such as chlorine RClS and bromine RBrS, although ROS and RNS are the two major groups involved in redox biology [6].

The superoxide anion is a primary oxygen radical that is formed when an oxygen molecule acquires an electron. The initial formation of O_2_^•−^ triggers a cascade of ROS, some of which, such as H_2_O_2_, behave as key molecules in cell signalling, and others, such as HO, are damaging. Ultimately, the biological impact of these molecules will be determined by the amount of ROS, cellular defences and the capacity for cellular adaptation [7].

O_2_^•−^ is one of the most important reactive oxygen species ROS responsible for oxidative stress in bio-organisms and is generated as a by-product of the mitochondrial respiratory chain [8]. Because of its charge, superoxide has a low membrane permeability, it passes through anion channels, but this is inefficient, and superoxide reacts to a large extent in the physiological compartment where it is generated.

**Figure 1:**
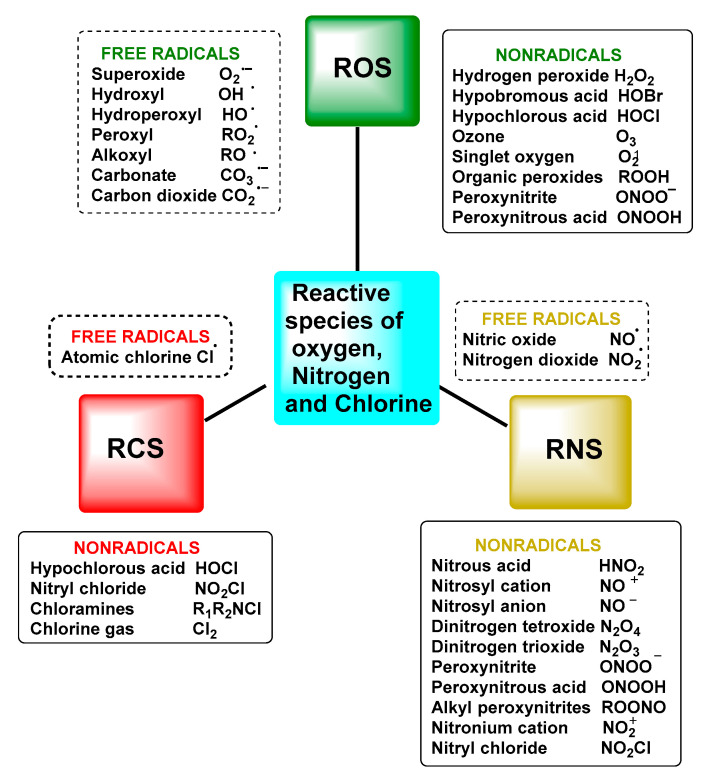
Nomenclature of reactive species and free radicals and other reactive oxygen, nitrogen and chlorine species.

Reactive oxygen species (ROS) are a group of highly reactive oxygen-containing chemicals produced exogenously or endogenously from the reduction of oxygen and include both radicals and non-radicals, one of which is superoxide. ROS present in the body are mostly of endogenous origin, although they can also be generated in response to external stimuli, such as ultraviolet light, ionising radiation, pollution, alcohol and tobacco consumption, drugs and toxic agents [9], Figure 1.

To control ROS, the body uses several antioxidant mechanisms, including enzymatic and non-enzymatic antioxidants [10]. Non-enzymatic low-molecular-weight antioxidant compounds include cellular glutathione, vitamins C and E, β-carotene, polyphenols and uric acid. Antioxidant enzymes include superoxide dismutase, catalase, glutathione reductase and glutathione peroxidase, among others. SOD catalyses the dismutation of superoxide to H_2_O_2_. Mammalian cells contain three forms of SOD: Mn-SOD, cytosolic Cu, Zn-SOD and extracellular Cu, Zn-SOD. MnSOD is most abundant in the mitochondria, whereas Cn, Zn-SOD predominates in the cytoplasm [11]. Catalase is an important antioxidant enzyme that catalyses the reduction of H_2_O_2_ to H_2_O. Glutathione peroxidase is another important enzyme for the decomposition of H_2_O_2_. Polyphenols, ingested regularly through the fruit and vegetable diet, are a large family of natural organic compounds characterized by multiple hydroxyl phenolic units, with a polyphenolic structure, (several hydroxyl groups on aromatic rings), including four main classes: phenolic acids, flavonoids, stilbenes and lignans [12]. Evidence and research to date supports the role of polyphenols in the prevention of cancer, cardiovascular and neurodegenerative diseases [13]. A significant part of their beneficial effects are based on the modulation of cell signalling pathways [14].

## 2. Superoxide Radical Anion O_2_^•−^

**Figure 2:**
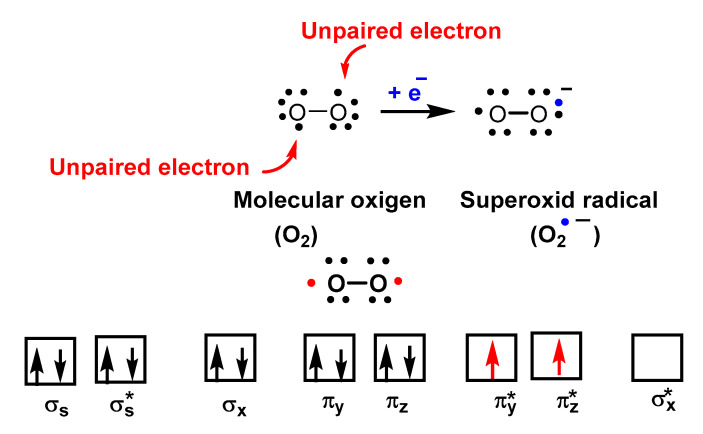
Molecular orbital diagram of O_2_ showing its biradical nature.

O_2_^•−^ is a reduced form of molecular oxygen O_2_, consisting of two oxygen atoms with 17 electrons and a negative electrical charge, Figure 2. Superoxide is the first species produced in the respiratory chain by the reduction of oxygen by the transfer of an electron and is one of the first species generated by various cellular systems. O_2_^•−^ is formed in all living aerobic organisms, and can act as a signalling agent, a toxic specie or a harmless intermediate that spontaneously decomposes. Its levels are limited in vivo by two different types of enzymes, superoxide reductase SOR and superoxide dismutase SOD.

**Figure 3:**
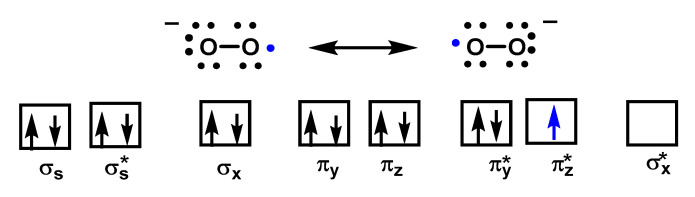
The molecular orbital of O_2_^•−^ shows one unpaired electron and is delocalized between the π* orbitals of the two oxygen atoms.

Despite being a “free biradical”, oxygen has a low reactivity because the unpaired electrons of each oxygen atom have parallel spins, Figure 3.

**Figure 4:**
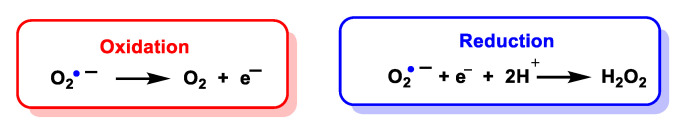
Oxidation and reduction of O_2_^•−^ to form oxygen or hydrogen peroxide, respectively.

Superoxide is considered both a radical and a −1 charged anion. It is a relatively unstable molecule, with a half-life of milliseconds, a reasonably strong oxidant, in which case it is reduced to hydrogen peroxide, and can also act as a reductant and convert to oxygen. There are two standard redox potentials for O_2_^•−^ showing that it can act as a reducing agent E′(O_2_/O_2_^•−^) = 0.33 V or as an oxidizing agent E′(O_2_^•−^/H_2_O_2_) = 0.93 V [15]), Figure 4.

**Figure 5:**
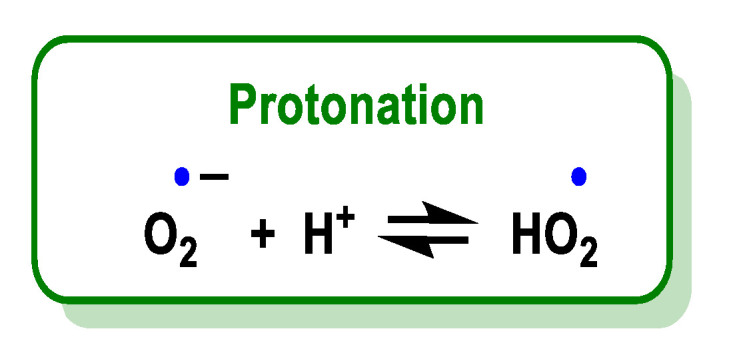
Protonation of O_2_^•−^ leads to the formation of HO_2_^•^.

O_2_^•−^ is a relatively small anion, highly soluble in water, where it is solvated by four water molecules strongly bound by hydrogen bonds [16] and reacts with a proton or proton donor to form HO_2_^•^, Figure 5. Various organic and inorganic compounds can act as a source of a proton in a large number of reactions [17].

The superoxide radical is the conjugate base of a weak acid, the hydroperoxide radical HOO^•^, whose pKa is 4.88 [18]. The pH controls the distribution between HO_2_^•^ and O_2_^•^.

Near the membrane, where this radical is produced, the pH is much lower than in the cytoplasm, so the acid form or hydroperoxide radical will predominate. Due to its non-ionic nature, it can enter the cell membrane and trigger lipid peroxidation processes [19]. The hydroperoxide radical is much more reactive, more oxidising than the superoxide radical, but in aqueous solution at physiological pH the non-protonated form, i.e., the superoxide radical, predominates. Perhydroxyl constitutes less than 1% of superoxide at neutral pH so its impact is more limited. Superoxide absorbs light in the ultraviolet range with a maximum at 245 nm and an extinction coefficient of 2350 M^−1^ cm^−1^, whereas hydroperoxyl absorbs at 225 nm with an extinction coefficient of 1400 M^−1^ cm^−1^ [20].

**Figure 6:**
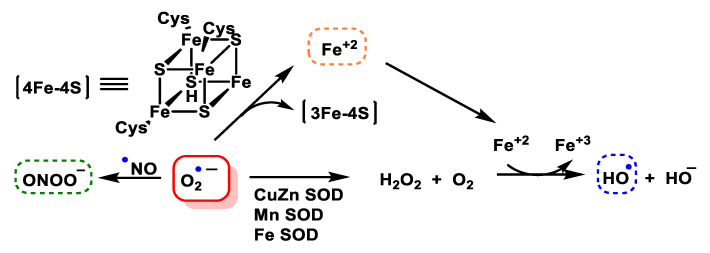
Generation of hydroxyl radical, peroxynitrite and hydrogen peroxide by the O_2_^•−^ anion.

O_2_^•−^ is toxic, mainly because it damages proteins containing Fe-S centres, such as aconitase, succinate dehydrogenase and NADH-ubiquinone oxidoreductase, among others. However, it can also be the generator of other reactive species even more toxic than itself, the iron released from iron and sulphur proteins can give rise to secondary products, such as hydroxyl radicals, and these, plus peroxynitrite, are thought to be the main contributors to superoxide toxicity. Superoxide dismutase SOD is the enzyme responsible for transforming this reactive species into one of a lower toxicity, such as hydrogen peroxide H_2_O_2_, Figure 6.

O_2_^•−^ reacts slowly with most molecular targets, although it has been shown to disrupt iron and sulphur group enzymes [21]. However, O_2_^•−^ can rapidly react with other radicals to give other reactive species [22].

## 3. Sources of Superoxide Anion

### 3.1. Biological Sources

**Figure 7:**
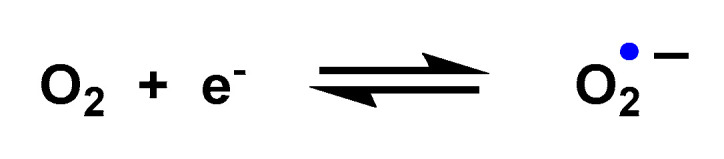
Oxygen reduction to O_2_^•−.^

Oxygen is an element that has a dual physiological effect; it is essential for the development of aerobic life and has toxic effects inherent to its structure. Oxygen utilisation by aerobic organisms, under normal conditions, generates reactive oxygen metabolites that can lead to a state of oxidative stress if the pro-oxidant/antioxidant cell balance is disturbed. Superoxide is a primary radical formed when an oxygen molecule acquires an electron through enzymatic or non-enzymatic reactions [11], Figure 7.

Under basal conditions, human cells produce about 2 trillion O_2_^•−^ and H_2_O_2_ per cell per day, the major source of which is the mitochondria [23]. These organelles consume 80-90% of cellular oxygen, in which they reduce water to obtain energy in the form of ATP. Although mitochondrial respiration is highly efficient, approximately 2% of the O_2_ consumed is partially reduced to O_2_^•−^ and H_2_O_2_.

**Figure 8:**
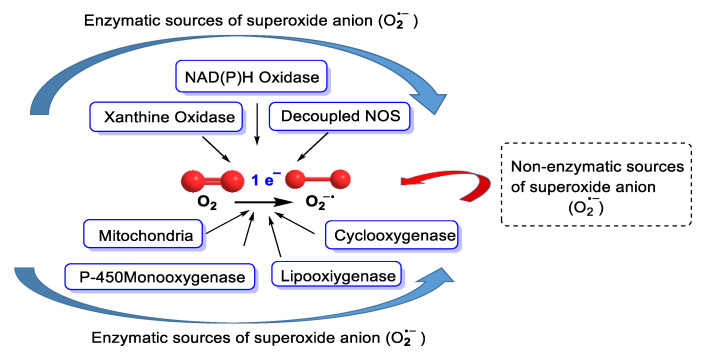
Enzymatic sources of superoxide anion and non-enzymatic production of superoxide [25].

Metabolic reactions that consume oxygen molecules are the main source of superoxide. Biologically, O_2_^•−^ can be generated from the mitochondrial electron transport chain (ETC), which is the main source of O_2_^•−^, and many enzymes, such as NADPH oxidase NOX, xanthine oxidase XO, lipoxygenase, cyclooxygenase, and cytochrome P450 CYP/cytochrome P450 reductase POR, and electron transport chains found in the endoplasmic reticulum, peroxisomes, nuclear membrane and cytoplasmic membrane, convert O_2_ to superoxide [24], Figure 8. Superoxide can also be produced non-enzymatically.

### 3.2. Mitochondrial Respiratory Chain

**Figure 9:**
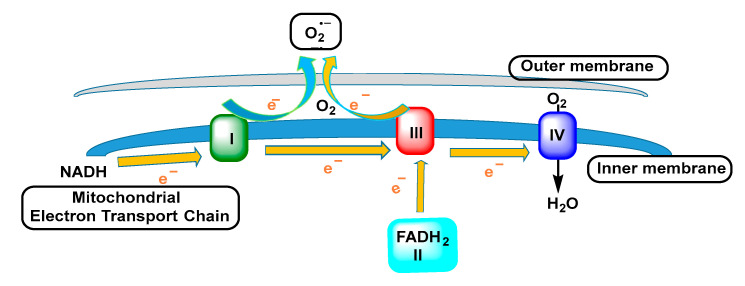
Superoxide radicals are produced in complexes I and III of the electron transport chain by transferring electrons to molecular oxygen.

The mitochondrion is the main producer of reactive oxygen species during the normal oxidative processes of metabolism, mainly through oxidation–reduction reactions occurring in electron transfer complexes with oxygen as the ultimate electron acceptor [26], Figure 9.

Complex I is the first multi-enzyme complex of the respiratory chain, with a central role in cellular energy production, being, in turn, one of the sites of generation of O_2_^•−^. The electron flow through the enzyme complexes in the inner membrane generates an electrochemical proton gradient and, therefore, produces energy. An undesired effect of the redox reactions occurring in mitochondria is the generation of reactive oxygen species [27].

**Figure 10:**
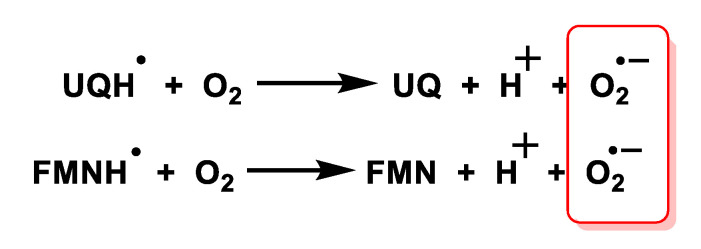
The mitochondrial production of superoxide radicals is carried out through two fundamental reactions: the oxidation of ubiquinol UQ and the autoxidation of flavin by FMNH dehydrogenase.

Mitochondria, present in all aerobic cells, are the most important biological source of superoxide carried out by two components of the mitochondrial respiratory chain, ubisemiquinone and the flavin semiquinone of NADH dehydrogenase. The superoxide radical is not able to cross the inner mitochondrial membrane so it is confined to the matrix where it reacts rapidly with the enzyme manganese-superoxide dismutase Mn-SOD and nitric oxide to form hydrogen peroxide and peroxynitrite, respectively [28], Figure 10.

### 3.3. NADPH Oxidases

NADPH oxidase in phagocytic cells produces large amounts of O_2_^•−^ in defence against pathogens and other aggressors [29].

**Figure 11:**
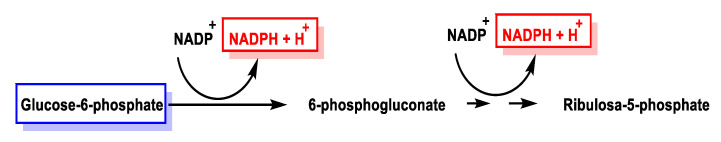
Formation of NADPH molecule in the transformation of glucose-6-phosphate into ribulose 5-phosphate.

The pentose phosphate pathway generates NADPH during the oxidative phase in which two NADP+ molecules are reduced to NADPH by utilising glucose-6-phosphate in ribulose 5-phosphate, Figure 11.

**Figure 12:**
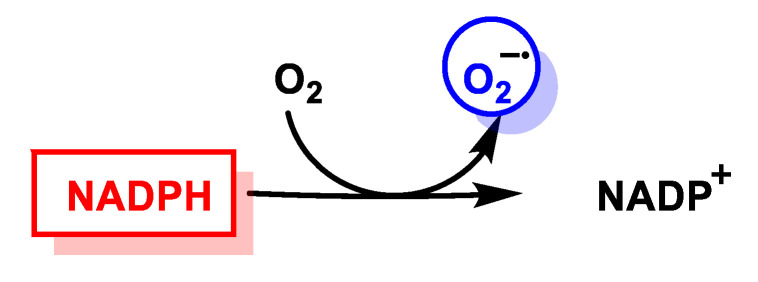
Reduction of O_2_ to O_2_^•−^ by NADPH.

NADPH subsequently reduces O_2_ to O_2_^•−^ via the NADPH oxidase pathway. In the rest of the non-phagocytic cells, NADP oxidase is represented by NOX (non-phagocytic NADPH oxidase), enzymes producing small constitutive pulses of O_2_^•−^, which are key players in cell signalling [30], Figure 12.

**Figure 13:**
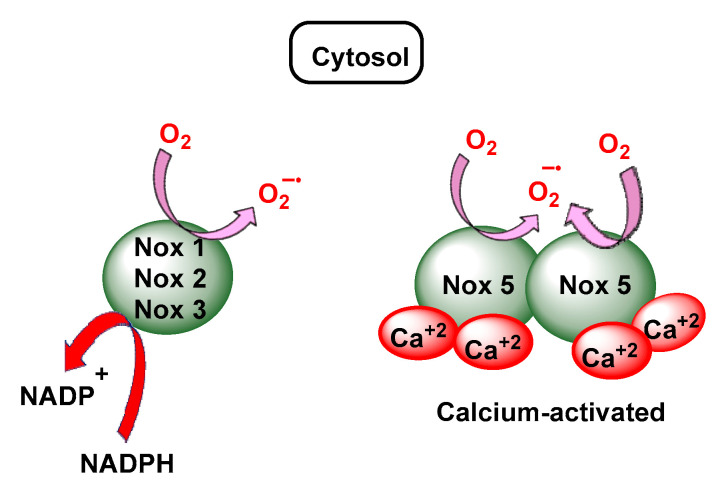
The NOX family of O_2_^•−^ generating NADPH oxidases.

NOX are mainly located in the plasma membrane [31,32]. NOX2 proteins are constitutively present and, upon inflammatory stimuli, activated NOX2 converts molecular oxygen to superoxide using electrons from NADPH and releases superoxide. NOX2 is predominantly expressed in phagocytes and produces a relatively large amount of superoxide in order to kill bacteria within phagosomes in an inflamed area [33,34], Figure 13.

Superoxide radicals, hydrogen peroxide, singlet oxygen and hypochlorous acid HOCl are generated in the cell membrane through the action of the enzymes NADPH oxidase, myeloperoxidase and xanthine oxidase [35,36]. Other enzymes, such as lipooxygenase and cyclooxygenase also generate ROS during the synthesis of leukotrienes, thromboxanes and prostaglandins [35].

### 3.4. Cytochrome P450 CYP/Cytochrome P450 Reductase POR System

**Figure 14:**
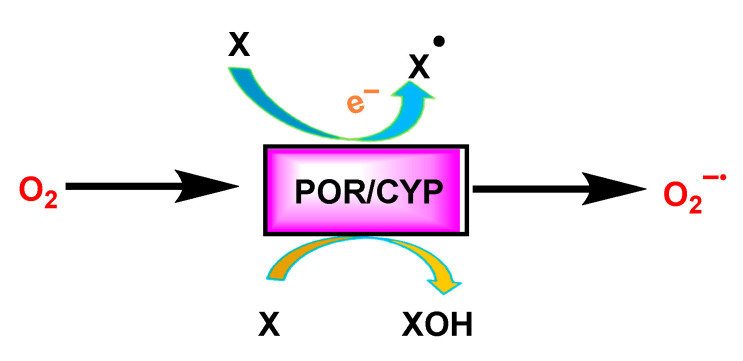
The enzymes cytochrome P450 (CYP)/cytochrome P450 reductase POR, convert molecular oxygen to superoxide either as a main product or as a by-product during oxidation of a variety of compounds X.

In the endoplasmic reticulum, superoxide radicals and hydrogen peroxide are produced by the auto-oxidation of the flavoprotein NADPH, cytochrome P450 reductase and cytochrome P450. In addition, mixed function monooxygenases provide another important source of superoxide. The CYP reaction together with POR releases superoxide as a by-product of the oxidase reaction [36], Figure 14.

### 3.5. Xanthine Oxidoreductase

**Figure 15:**
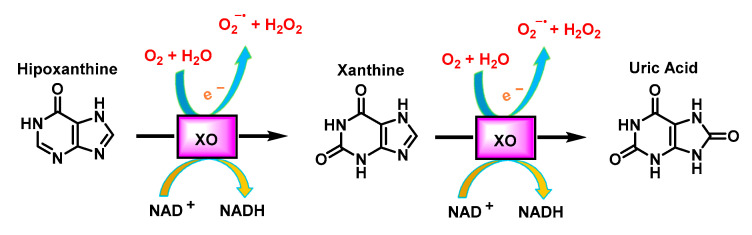
Reactions catalysed by xanthine oxidase.

Xanthine oxidase XO uses oxygen molecules, instead of NAD+, as an electron acceptor and produces superoxide or hydrogen peroxide. It is a cytosolic metalloflavoprotein that can be in two interconvertible and distinct forms called xanthine dehydrogenase XDH and xanthine oxidase XO. XO belongs to a family of molybdo-flavoenzymes and is released by a calcium-activated protease during hypoxia. XO is unique in generating superoxide (28%) and H_2_O_2_ (72%) by oxidation of hypoxanthine to xanthine, and xanthine to uric acid, Figure 15. XO activity is increased in inflammatory airway disorders, ischaemic reperfusion injury, atherosclerosis, diabetes and in autoimmune disorders [37].

The first two processes involve the reduction of two electrons from O_2_ to form H_2_O_2_, then the remaining two electrons are each used to reduce O_2_ to O_2_^•−^. The total ROS produced is therefore two H_2_O_2_ and two O_2_^•−^ molecules.

### 3.6. Non-Enzymatic Production of Superoxide

**Figure 16:**
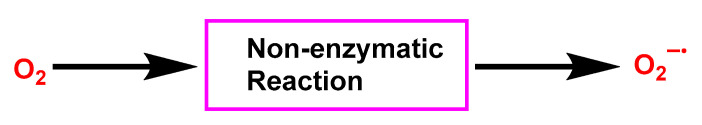
Catalysed reactions by Xanthine Oxidase.

Superoxide can also be produced by a number of non-enzymatic reactions [25]. Above all, non-enzymatic glycosylation, referred to as glycation, occurs under conditions of hyperglycaemia and produces a variety of compounds [38,39], Figure 16.

### 3.7. Non-Biochemical Sources

**Figure 17:**
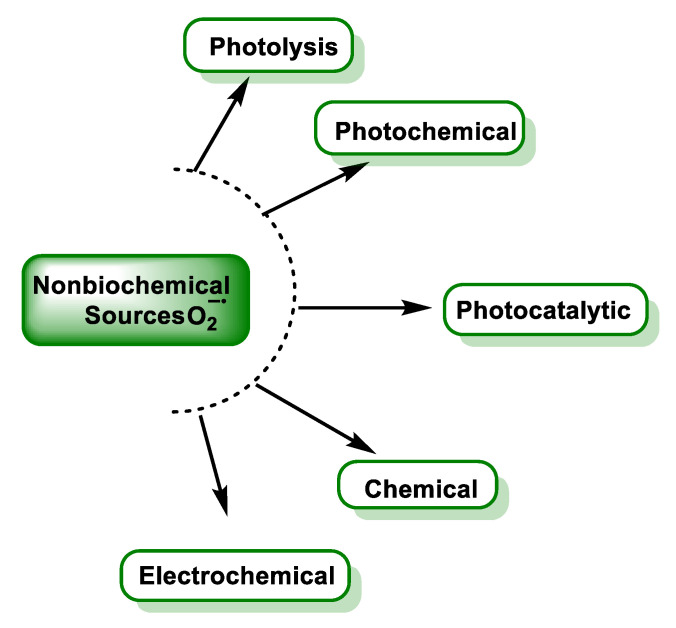
Non-Biochemical sources of superoxide [25].

Several techniques to produce superoxide have been used to study its reactions. Chemically, the main ways to produce superoxide are reactions involving ionising radiation or UV, and O_2_ reduction by transition metals or reducing radicals, Figure 17.

### 3.8. Photolysis

**Figure 18:**
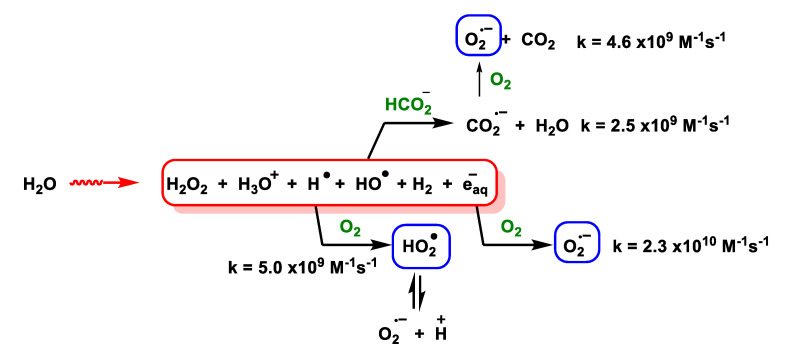
The radiolysis of water is performed under high-energy electrons and if the water contains molecular oxygen and sodium formate, the primary radicals become O_2_^•−^/HO_2_^•^.

For the generation of O_2_^•−^ during the ionising irradiation of air-saturated sodium formate, an in-line stopped-flow radiolysis apparatus with a Van de Graaff electron generator has been used to generate O_2_^•−^ during the ionising irradiation of sodium formate saturated with air at 2 MeV [40], Figure 18.

**Figure 19:**
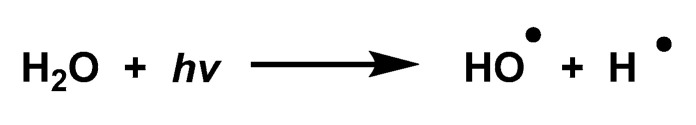
Photochemical decomposition of water through its dissociation into HO^•^ and H^•^.

Superoxide can also be formed in an O_2_-saturated aqueous formate solution after brief UV irradiation with a Xe or Ar lamp. The main process involves the photochemical decomposition of water through its dissociation into HO^•^ y H^•^, Figure 19.

**Figure 20:**
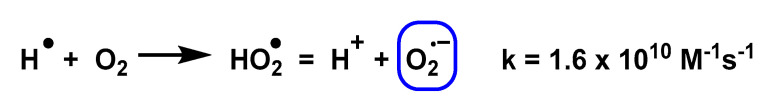
Reaction of H^•^ with oxygen to form O_2_^•−^.

**Figure 21:**
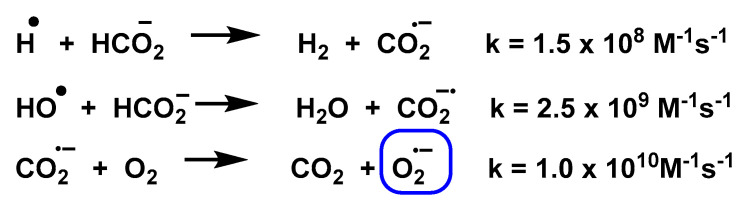
The CO_2_^•−^ formed reduces the O_2_ to O_2_^•−^.

In the presence of O_2_, electron transfer occurs to produce O_2_^•−^. In addition, formate HCOO^–^ can react with H^•^ or HO^•^ to form a common product CO_2_^•^, where CO_2_^•^ can further reduce O_2_ to O_2_^•−^, Figure 20 and Figure 21.

**Figure 22:**
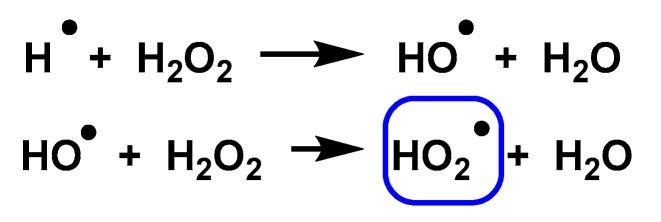
UV photolysis of H_2_O_2_ forms HO_2_^•^.

Photolysis of an H_2_O_2_ solution can also convert HO^•^ and H^•^ to HO_2_^•^ through the reactions shown in Figure 22.

### 3.9. Photochemical and Photocatalytic Sources

O_2_^•−^ generation can be initiated photochemically by electron transfer [41]. Aqueous and ethanolic alkaline superoxide solutions can be prepared by vacuum UV photolysis or high-energy ionising radiation and are long-term stable and can be stored at low temperatures. There are three main pathways for the photochemical generation of O_2_^•−^ and all three pathways require O_2_ for O_2_^•−^ production.

**Figure 23:**
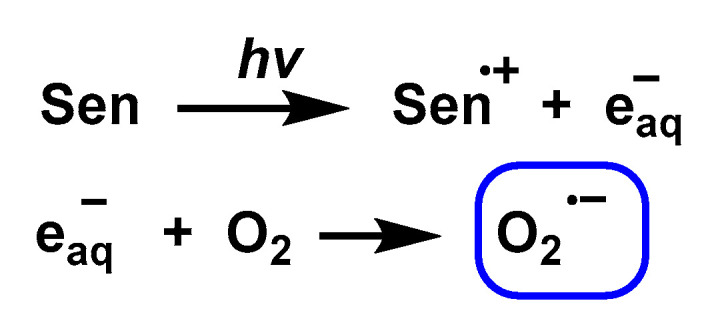
The hydrated electron (e_aq_^−^) directly reduces O_2_ to O_2_^•−^.

Pathway 1. Photoionisation of a sensitising molecule that generates a hydrated electron (e_aq_-) which in turn directly reduces O_2_ to O_2_^•−^, Figure 23.

Tryptophan and other amino acids, aromatic compounds (such as amines, phenols, methoxybenzenes and indoles) are able to generate O_2_^•−^ under photoionisation conditions in near ultraviolet light.

**Figure 24:**
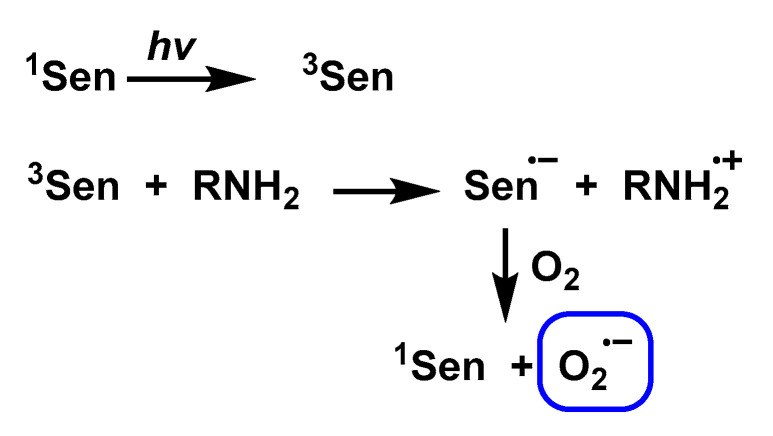
Reduction of O_2_ by Sen^•−^ to form O_2_^•−^.

Pathway 2. Use of an excited state acceptor (Sen) that can accept electrons from a ground-state electron donor, such as an amine or other electron-rich substrates to form Sen^•−^, which then leads to the reduction of O_2_ by Sen^•−^ to form O_2_^•−^, Figure 24.

This pathway 2 involves a charge transfer mechanism that is very common between flavin and its analogues and generally occurs in the presence of an electron donor, such as EDTA. Methylene blue in the triplet state in the presence of alkylamines results in the transfer of electrons to generate O_2_^•−^.

**Figure 25:**
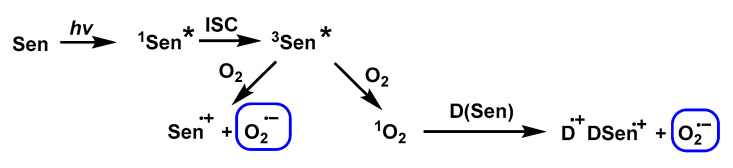
The formation of O_2_^•−^ from ^1^O_2_ using specific ^1^O_2_ inhibitors.

Pathway 3. Electron transfer with O_2_ or ^1^O_2_ by a sensitised ground-state or excited-state donor, respectively. Mechanism 3 shows the formation of O_2_^•−^ from ^1^O_2_ (^1^O_2_ is generated from O_2_ sensitisation by rose bengal) using furfuryl alcohol [42], fullerenes [43] or quinones [44] as specific inhibitors of ^1^O_2_, Figure 25.

In photocatalysis, electrons react with molecular oxygen through the reductive pathway to generate O_2_^•−^ [45,46,47,48]. Goto et al., 2004, in the study of photocatalysed reactions with titanium dioxide TiO_2_ using aqueous solutions containing 2-propanol, found that O_2_^•−^ was the main product when rutile particles were used [49]. O_2_^•−^ can also be photogenerated by Bi 2+ x WO 6 (x = 0, 0.05, 0.1, 0.15 and 0.2) under visible light irradiation with autodoping [50].

### 3.10. Chemical Pathway

The chemical generation of O_2_^•−^ consists of two main steps:

- Synthesis of superoxide salts of alkali metals, such as potassium and sodium, and alkali earth metals, such as strontium and barium.
- Solvation of these salts in appropriate media to release O_2_^•−^.

Superoxide ions can be generated by dissolving superoxide salts in aprotic solvents. Several studies have been carried out and it has been found that the solubilities of KO_2_ and NaO_2_ in DMSO are extremely low and they are almost insoluble in all other organic solvents [51,52,53]. However, their solubilities are increased by the addition of tetraalkylammonium salts, such as tetramethylammonium superoxide [54,55]. Na_2_O has been found to be less reactive than K_2_O due to its reduced solubility [56]. The generation of O_2_^•−^ from its salts could be the best method for some industrial processes, such as the destruction of chlorinated hydrocarbons (CHCs).

### 3.11. Electrochemical

The electrochemical method provides a source of O_2_^•−^. The electrochemical generation of O_2_^•−^ by reduction of O_2_ is a good method because it generates no by-products. The procedure is relatively simple, is time efficient and has been used to study the kinetics of O_2_^•−^ reactions with other substances. Apotic solvents are used. Using the standard O_2_/O_2_^•−^ potential, E_0_ = −0.284 V, O_2_^•−^ can be generated electrochemically from the one-electron reduction of O_2_ in an alkaline aqueous solution in DMSO [57] using ionic liquids and some supporting electrolytes with aprotic solvents, such as tetra-n-butylammonium perchlorate, tetramethylammonium perchlorate, tetraethylammonium perchlorate, tetra-n-butylammonium iodide [58,59,60,61] and tetraethylammonium tetrafluoroborate [62]. Electrochemical generation of O_2_^•−^ is more favourable for some processes, in particular those involving fine chemicals and biological systems requiring ultra-high purity.

## 4. Reactions of Superoxide Anion

The superoxide is the lead radical that provides or removes an electron from other compounds, resulting in the production of other radical species through a chain reaction until the electron radical is finally removed. Superoxide oxidises few biological compounds [20], and this is due to the following reasons:

- Its anionic charge which limits its reactivity to compounds with electron-rich centres [63].
- Its facility for spontaneous dismutation [64].

Superoxide is a selective oxidant, relatively unreactive with most cell components, but highly reactive with some essential sites and therefore highly toxic. Superoxide ions are capable of undergoing several reactions, including disproportionation, one-electron transfer, deprotonation and nucleophilic substitution [65,66,67].

### 4.1. Dismutation of Superoxide to Hydrogen Peroxide

#### 4.1.1. Non-Enzymatic Spontaneous Dismutation

**Figure 26:**
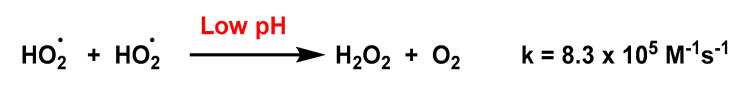
Disproportionation reaction of HO_2_^•^ and rate constant.

A high percentage of the superoxide will undergo rapid disproportionation to give hydrogen peroxide and oxygen [64]. O_2_^•−^ and HO_2_^•^ are reductants in a wide variety of reactions, but for most reactions only HO_2_^•^, and not O_2_^•−^, is an oxidant because of the need for a proton or a coordinated metal ion to stabilise the peroxide dianion, O_2_^=^, as it forms. This explains the pH dependence of the spontaneous disproportionation of superoxide. At very low pH, the predominant species is HO_2_^•^, an uncharged species, which acts as both a reductant and an oxidant, Figure 26.

**Figure 27:**
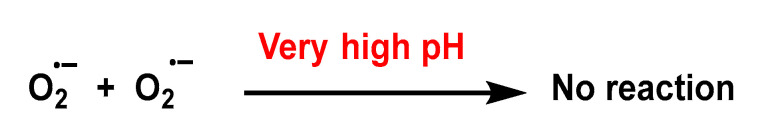
At very high pH, the superoxide anion does not disproportionate.

At very high pH, the predominant species is O_2_^•−^ itself, and it is quite stable under these conditions. The two superoxide anions repel each other and O_2_^=^ is unstable, so the disproportionation reaction does not take place, Figure 27.

**Figure 28:**
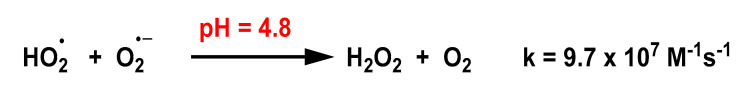
Disproportionation reaction of O_2_^•−^ and HO_2_^•^ and rate constant.

The disproportionation reaction is fastest at pH = pKa = 4.8, where the concentrations of HO_2_^•^ and O_2_^•−^ are equal, the former acting as an oxidant and the latter as a reductant, Figure 28.

O_2_^•−^ is not able to oxidise most organic substrates, including peptides, nucleic acids, lipids and carbohydrates, due to the requirement for a proton to stabilise O_2_^•−^ as it is formed at rates that compete with the disproportionation of superoxide in aqueous solution. Exceptions are substrates, such as ascorbate or hydroquinone, that have hydrogen atoms available.

#### 4.1.2. Enzymatically Catalysed Dismutation

**Figure 29:**
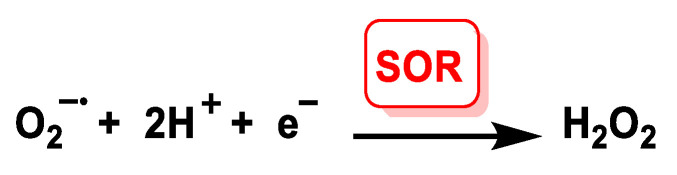
ROS catalyse the one-electron reduction of O_2_^•−^ to H_2_O_2_.

The rate of spontaneous non-enzymatic dismutation is relatively low (at physiological pH, about 2 × 10^5^ M^−1^ s^−1^). Catalysis of the dismutation reaction by the enzyme superoxide dismutase increases this rate in the order of 10 000-fold. Under normal circumstances, the biological system releases an enzyme, superoxide dismutase SOD, which specifically maintains the O_2_ concentration at an optimal level of 10^–10^ M [68]. Superoxide dismutases are a family of enzymes present in all aerobic cells that function by efficiently catalysing the dismutation of superoxide anions. Most of the superoxide produced physiologically undergoes SOD-catalysed dismutation. SORs and SODs are redox-active metalloenzymes that reduce superoxide concentrations. SORs catalyse the one-electron reduction of O_2_^•−^ to give H_2_O_2_, in this reaction two protons per mole of superoxide are required, and the presence of an external reductant is necessary to provide the electron, Figure 29.

**Figure 30:**
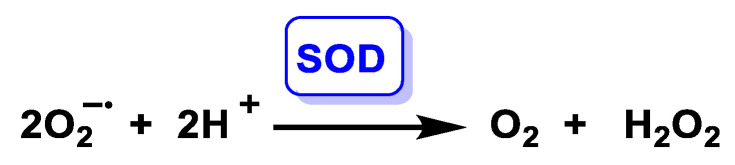
SOD disproportionation of superoxide to O_2_ and H_2_O_2_.

SOD catalyses the disproportionation of superoxide to give O_2_ and H_2_O_2_, a reaction that requires one proton per mole of superoxide, but no external reductant. The difference between the three proteins lies in their cellular location and regulation, which leads to differences in the source of the superoxide they will detoxify, as well as the time at which their action is required, Figure 30.

An important difference between SORs and SODs is that SORs contain only iron. OR and SOD enzymes have the ability to react selectively with superoxide in both the oxidised and reduced SOD and SOR states [69]. In particular, the reduced enzymes are rapidly oxidised by superoxide but not by O_2_, and the oxidised forms of SOD are rapidly reduced by superoxide. SOD enzymes catalyse the disproportionation of O_2_^•−^ by a very similar ping-pong mechanism, in which O_2_^•−^ acts alternately to reduce the oxidised metal ion and then to oxidise the reduced metal ion, the ping-pong mechanism requiring the enzyme’s reduction potential E° to lie between the oxidation and reduction potentials of superoxide. SOR enzymes perform only the latter of these two steps; that is, O_2_^•−^ performs the Fe^2+^ oxidation step but not the Fe^3+^ reduction step.

Superoxide dismutase SOD is the enzyme responsible for transforming this reactive species into a less toxic one, such as H_2_O_2_, which is subsequently transformed into water by other enzymes. All aerobic species have developed multiple strategies to detoxify ROS. The first line of defence is the dismutation (transformation) of superoxide to hydrogen peroxide by the enzyme SOD which catalyses a dismutation reaction where one molecule of O_2_^•−^ is oxidised to molecular O_2_, while the other is reduced to H_2_O_2_. The enzyme superoxide dismutase is the cell’s first enzymatic defence against superoxide radical production. To generate hydrogen peroxide and oxygen, this enzyme catalyses the dismutation of the superoxide radical. In mammals, this family consists of three members, which are located in clearly specific places, two inside the cell and one extracellular. In the cytoplasm, there is SOD1 or Cu, Zn-SOD [70], which contains copper and zinc in its catalytic centre. The second enzyme is located near the mitochondrial inner membrane. This is a SOD that binds manganese in its catalytic centre (SOD2 or Mn-SOD) [71,72]. Finally, there is one more, called SOD3 or EC-SOD [73], located outside the cell (in the extracellular matrix), and has copper and zinc and is the only extracellular protein that can remove O_2_^•^. The first and most obvious similarity between these enzymes is that they all contain redox-active metal ions in their active sites: Fe^2+/3+^ in Fe-SOD and SOR, Mn^2+/3+^ in Mn-SOD and Cu^1+/2+^ in Cu,Zn-SOD. Although all three perform the same catalytic activity, they differ greatly in their structure and organisation.

**Figure 31:**
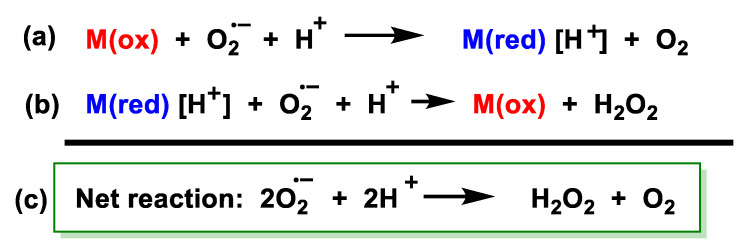
SOD-mediated reaction mechanism: (**a**) elimination of the first superoxide anion and reduction of copper; (**b**) second part of the reaction of transformation of the second peroxide anion into hydrogen peroxide and regeneration of the catalytic centre; (**c**) net reaction. Where M(ox) is the oxidized state of the metal in the catalytic centre (Cu^2+^, Zn^2+^ or Mn^3+^), and M(red) denotes the reduced state of the same metal (Cu^+^, Zn^2+^ or Mn^2+^).

The enzymatic reaction is carried out by SOD in two steps, both of which are first-order enzymatic reactions with respect to O_2_^•−^, Figure 31.

#### 4.1.3. Copper, Zinc-Superoxide Dismutase (Cu,Zn-SOD)

**Figure 32:**
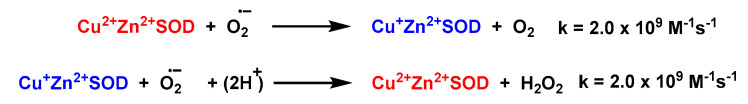
Superoxide dismutation reaction catalysed by Cu,Zn-SOD.

Several studies have been carried out using pulsed radiolysis to generate HO_2_^•^/O_2_^•−^ and to understand the Cu,Zn-SOD-catalysed reaction of superoxide dismutation [74,75,76,77], Figure 32.

A detailed examination of the first reaction using a pulse radiolysis technique showed that the enzymatic reduction and oxidation of superoxide were independent of pH. However, the second reaction was pH-dependent [78].

**Figure 33:**
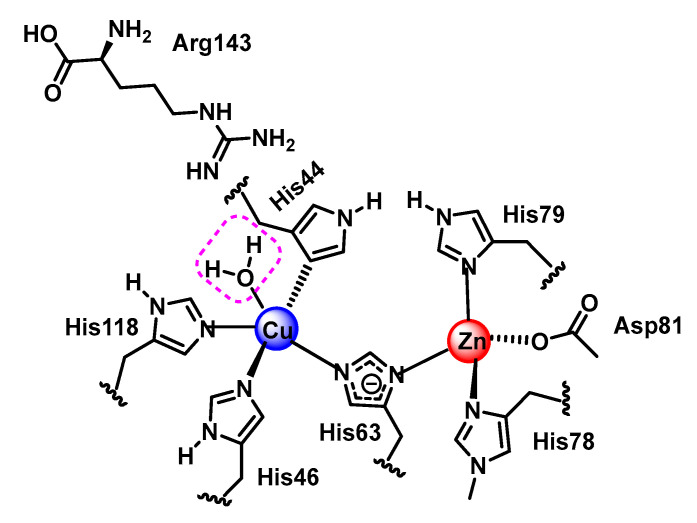
SOD1 catalytic centre.

Cu,Zn-SOD is a dimeric enzyme with each monomeric unit containing a copper and zinc active site linked by a histidine imidazole. Copper is bound by three additional histidines with a distorted square planar structure and an additional water molecule. Zinc is bound by two histidines and an aspartate in addition to the imidazole bridge. Copper is the redox active metal and zinc appears to play a role in the overall stability of the enzyme and in facilitating a high-pH-independence of enzyme activity [79], Figure 33.

**Figure 34:**
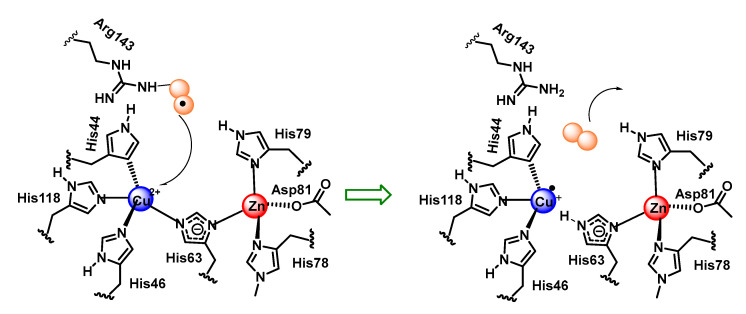
First phase of the reaction mediated by SOD1 with elimination of O_2_^•−^ reduction of copper and formation of O_2_.

The reaction mechanism of SOD1 unfolds as follows: O_2_^•−^ arrives at the reaction centre and binds, by an electrostatic interaction, to arginine 143. O_2_^•–-^ needs to donate its unpaired electron in order to be converted to molecular oxygen, as it is this electron that gives it its reactive and toxic nature. The electron is transferred to Cu^2+^, which transforms the metal to its lower oxidation state, or Cu^+^. This electron transfer causes the bond between Cu and histidine 63 to be broken, which causes the nitrogen in histidine 63 to be protonated. The O_2_ formed dissociates from arginine 143 and is released, Figure 34.

**Figure 35:**
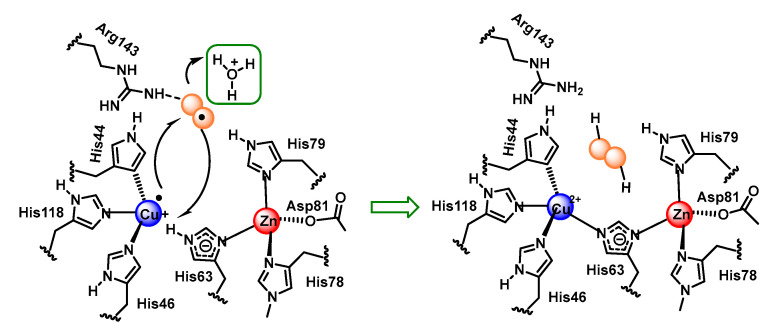
Second part of the reaction mediated by SOD1, transformation of the second O_2_^•−^ into hydrogen peroxide and regeneration of the catalytic centre.

The second part of the reaction starts in a similar way to the first part, the O_2_^•−^ arrives at the catalytic centre, and once there, it binds by an electrostatic interaction with arginine 143. Together with this, the protonation of a water molecule H_3_O^+^ is generated in the vicinity of the catalytic centre. The electron that Cu received in the first part of the reaction is now transferred to the second O_2_^•−^ allowing the oxidation of the metal to Cu^2+^. The two electrons now possessed by the superoxide can immediately form two covalent bonds with two protons, which are donated, one by the protonated water molecule, and the other by the nitrogen of histidine 63, thus re-establishing the bond it originally formed with Cu^2+^. This allows the release of H_2_O_2_ and the regeneration of the enzyme, Figure 35.

#### 4.1.4. Manganese Superoxide Dismutase Mn-SOD

**Figure 36:**
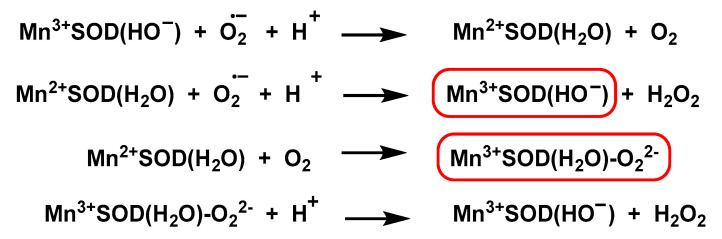
The mechanism includes the formation of both Mn^3+^SOD and the complex (Mn^3+^ SOD(H_2_O)-O_2_^2−^).

In the Mn-SOD structure, manganese binds to four protein ligands (three histidines and an aspartic acid residue) and a fifth solvent ligand in a trigonal bipyramidal geometry. The active sites and structures of three MnSODs (human, *Escherichia coli*, and *Deinococcus radiodurans*) have been studied in detail [80,81,82]. The mechanism of Mn-SOD dismutation is complex [83]. Using pulse radiolysis studies, the following mechanism has been proposed [84,85], Figure 36.

#### 4.1.5. Iron Superoxide Dismutase Fe-SOD

**Figure 37:**
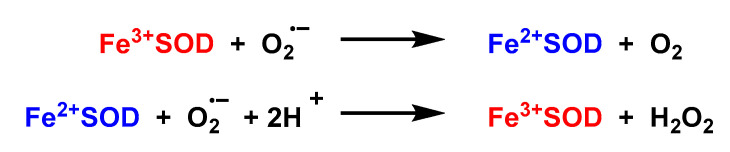
A ping-pong mechanism is displayed in the dismutation of O_2_^•−^ by FeSOD.

It is a prokaryotic enzyme, discovered in some bacterial cells and in the cytosol of plants [86]. Most of the structural and mechanistic studies have been performed on Fe-SOD obtained from *E. coli*. The structures of Fe-SOD are dimers. The active site contains a single iron atom bound to three histidines, an aspartate and a water molecule [87]. The coordinated water molecule involves a hydrogen bond with an aspartate ligand and another with the conserved active site ligand, glutamine 69. A ping-pong mechanism in the dismutation of O_2_^•−^ by Fe-SOD is proposed [88], Figure 37.

#### 4.1.6. Iron Superoxide Reductase Fe-SOR

SORs are small enzymes, with about 110–180 amino acids in their sequences. Based on the number of metal centres, there are two types of Fe-SORs: neelaredoxins (1Fe-SORs) and desulfoferrodoxins (2Fe-SOR) [89]. In Fe-SOR, the iron is close to the molecular surfaces and is exposed to the solvent. The metal centres in Fe-SODs are located inside the protein. Desulfoferrodoxins are a homodimeric non-heme iron protein found in some sulphate-reducing bacteria and archaea [90,91]. Neelaredoxins, from *Archaeoglobus fulgidus*, have biofunctional properties as SOD and ROS. Desulfoferrodoxins and neelaredoxins have been isolated mainly in the Fe^2+^ and Fe^3+^ forms, respectively. UV-visible spectroscopy has been applied to study the catalytic activity of SOR [92,93,94,95,96,97,98,99].

#### 4.1.7. Analytical Determination of Superoxide Dismutase Activity

**Figure 38:**
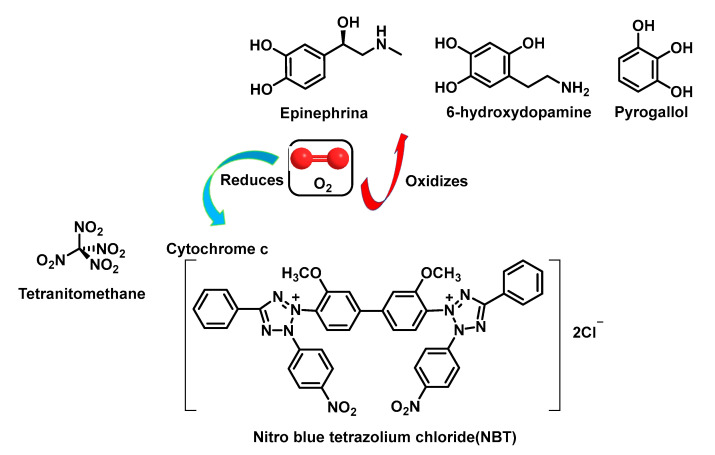
O_2_ as a monovalent oxidant or reductant.

O_2_ can act as either an oxidant or a monovalent reductant. This dual reactivity has been exploited in the design of assays to analyse SOD activity. Thus, in some assays, Oz reduces tetranitro methane [11], cytochrome c or nitro tetrazolium blue [100], and in others it oxidises epinephrine, tiron pyrogallol or 6-hydroxydopamine [101,102], Figure 38.

The measurement of SOD activity presents a major problem due to the instability of the superoxide radical in aqueous media. Direct determination of SOD activity is carried out by observing the disappearance of the free superoxide radical catalysed by this enzyme, generated by a pulse of electrons on a millisecond scale. This assay is not affordable for most laboratories as it requires the presence of a linear electron accelerator, so that determinations of SOD activity have had to rely on variations in constant concentrations of superoxide radicals catalysed by the enzyme. The most used methods require two components: (a) a superoxide radical generator and (b) a detector of the superoxide radical [103].

**Figure 39:**
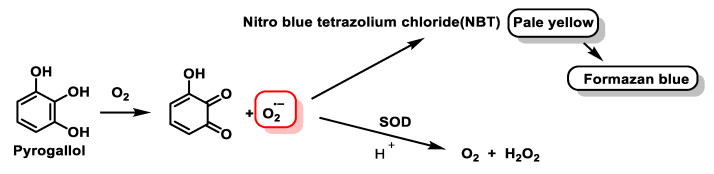
Generation of the superoxide radical by the chemical autoxidation of pyrogallol.

The generator produces the superoxide radical at a constant controlled rate. In the absence of SOD, the superoxide radical accumulates to such a concentration that the rate of reaction with the detector is equal to the rate of production, and this equilibrium state is reached within one second. If SOD is present, it competes with the detector for the superoxide radical, resulting in a decrease in the superoxide radical taken up by the detector, with an inhibition of the detection level. In the method described by Minami and Yoshikawa, 1979, the generation of the superoxide radical is produced by the chemical autoxidation of pyrogallol at pH = 8.2 [104], Figure 39.

**Figure 40:**
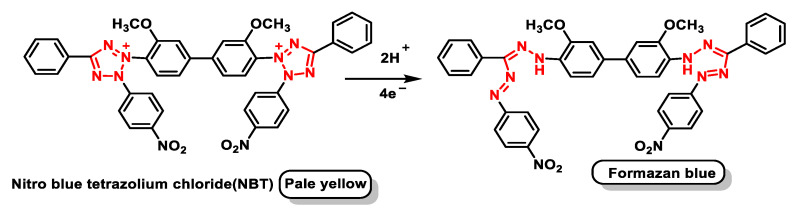
The reduction of NBT gives a compound with an intense blue colour, formazan blue.

The detector for this radical is a dye, NBT, a yellow compound which, in the presence of superoxide radical, is reduced, giving an intensely blue compound, formazan blue, Figure 40.

In this method, therefore, the inhibition of NBT reduction is measured, which is measurable spectrophotometrically. This inhibition is measured against a control in which there is no SOD. A unit of SOD activity is considered to be the activity of this enzyme that would produce 50% of the maximum inhibition caused by this enzyme on the reduction of NBT, according to the definition of McCord and Frldovich [105].

The NBT method has several disadvantages, such as the poor water solubility of the formazan dye and the interaction with the reduced form of xanthine oxidase. The replacement of NBT by Dojindo’s highly water-soluble tetrazolium salt WST-1 (2-(4-iodophenyl)-3-(4-nitrophenyl)-5-(2,4-disulphophenyl)-2H-tetrazolium, monosodium salt) overcomes these problems and makes it a very convenient SOD assay, yielding a stable water-soluble formazan dye with a high absorbance at 450 nm after reduction with a superoxide anion. The rate of reduction with O_2_^2-^ is linearly related to the activity of xanthine oxidase (XO) and is inhibited by SOD. Therefore, the IC50 (50% inhibition activity of SOD or SOD-like materials) can be determined by a colorimetric method.

**Figure 41:**
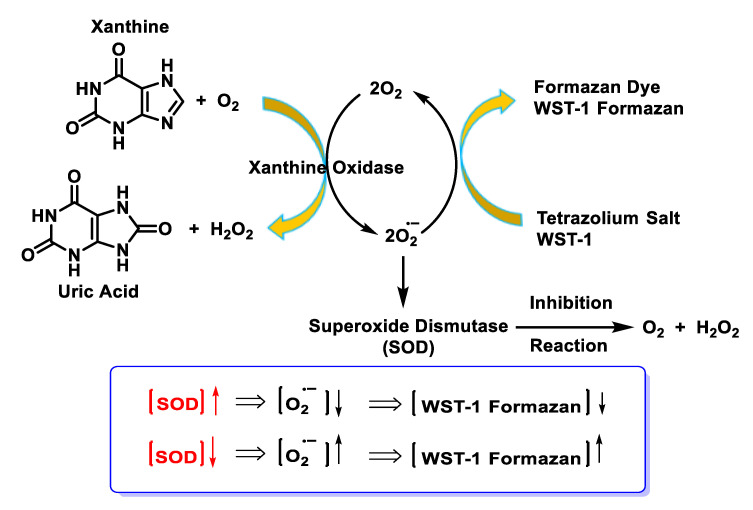
SOD inhibition assay mechanism.

Superoxide ions are generated from the conversion of xanthine and O_2_ to uric acid and H2O2 by xanthine oxidase (XO). The superoxide anion then converts the tetrazolium salt WST-1 to the coloured product WST-1 formazan [106], Figure 41.

**Figure 42:**
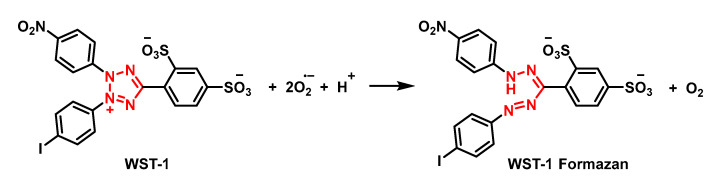
Structure of WST-1 (4-(3-(4-iodophenyl)-2-(4-nitrophenyl)-2H-tetrazol-3-ium-5-yl) benzene-1,3-disulfonate).

The absorbance is then measured at 450 nm using a standard microplate reader. The addition of SOD to this reaction reduces the levels of superoxide ions, which reduces the rate of WST-1 formazan formation, Figure 42. The SOD activity in the experimental sample is measured as the percentage inhibition of the rate of WST-1 formazan formation.

#### 4.1.8. Reaction with Iron–Sulphur [Fe–S] Cluster

The best-established detrimental effect of superoxide is the inactivation of iron/sulphur protein groups, such as aconitase, and bacterial dehydratases required for branched-chain amino acid synthesis. Mammalian aconitases (mitochondrial and cytosolic isoenzymes) are unique iron/sulphur-group-containing proteins in which the metal centre participates in the catalysis of a non-redox reaction. The [4Fe-4S] group exists in a cubic structure, with iron and sulphur atoms found in alternating corner positions. These groups are found in bacterial ferredoxins and within mitochondrial respiratory complexes. The detrimental effect of superoxide is the inactivation of iron/sulphur-containing protein groups. These reactions have rate constants in excess of 10^6^ M^−1^ s^−1^ and are highly selective for the superoxide [107]. Aconitase is an essential enzyme, particularly sensitive to oxidative damage and is preferentially modified and inactivated by mitochondrial oxidants during ageing and in pathologies involving mitochondrial dysfunction. It is one of the main targets of superoxide. Aconitase activity is also sensitive to nitric oxide, peroxynitrite and the carbonate radical [108].

Aconitase deficiency is associated with myopathies and low exercise endurance [109]. Certain variants of aconitase have been found to cause infantile cerebellar-retinal degeneration syndrome, which is characterised by various neurological and muscular symptoms [110].

**Figure 43:**
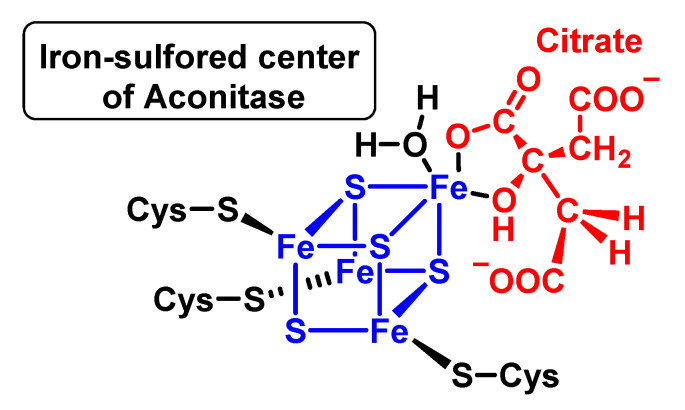
Iron–sulphur centre of aconitase and union with citrate and a water molecule.

Within the Cuban group of iron–sulphur aconitases, only three of the four iron ions have cysteine thiolate ligands; the fourth iron ion (Feα) is exposed to the solvent within the active site pocket and binds to the oxygen atoms of the water or substrates to be dehydrated. An example of binding of the ferro-sulphurised centre of aconitase with citrate is shown in Figure 43.

**Figure 44:**
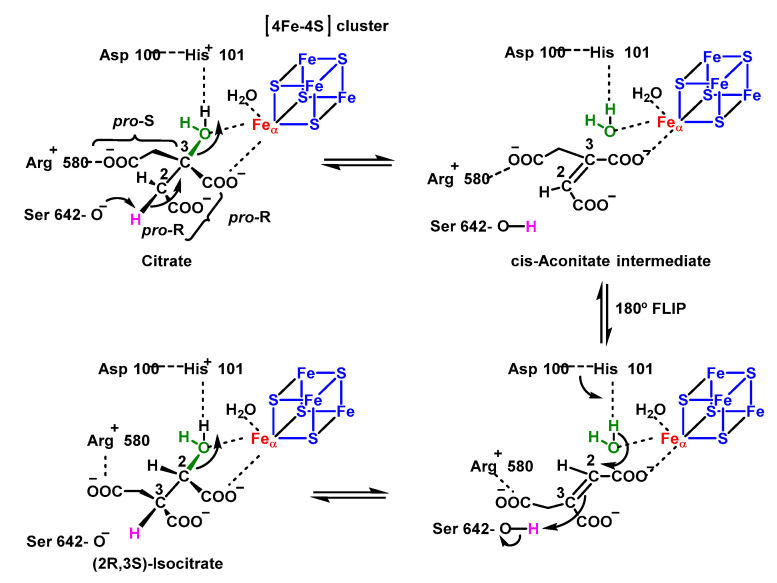
Mechanism of isomerisation of citrate to isocitrate by the enzyme aconitase. The aconitase differentiates between the two carboxylic groups of the citrate uniting it by three points.

Mitochondrial aconitase is a ferrosulphoprotein located in the mitochondrial matrix that catalyses the reversible isomerisation of citrate to isocitrate via cis-aconitate in the Krebs cycle [111], Figure 44. The reaction mechanism can be divided into three phases, a dehydration phase, followed by a rotation and finally a rehydration phase. In dehydration, the OH group attached to the Fe-S centre is protonated and eliminated, forming cis-aconitate. In order to rehydrate, the molecule needs to rotate 180º, which takes place in more than one step, a cis-aconitate molecule displaces the one it is attached to, and once it is properly attached, it is rehydrated, forming the final product.

This aconitase-catalysed reaction is stereospecific. Isocitrate has two asymmetric carbons, so that from citrate the formation of four possible stereoisomers would be possible, but the reaction produces only one of these, D-isocitrate. The enzyme has an asymmetric binding site for citrate, allowing the substrate to bind only in a specific orientation, so the transfer of the OH occurs only if the appropriate orientation conditions are met [112].

The high reactivity of aconitase with superoxide ion is due to the presence of the [4Fe-4S] centre with labile Fe in its active site [113]. The mechanism involves (i) electrostatic attraction of the superoxide to the solvent-exposed group, (ii) protonation to become a strong univalent oxidant, and (iii) single electron abstraction. The reactivity is attributed to the electrophilic character of Fe, the large nucleophilicity of O_2_^•−^, the increased oxidative potential of O_2_^•−^ in its iron-bound state and the sensitivity of Fe-S bonds to oxidation [113].

**Figure 45:**
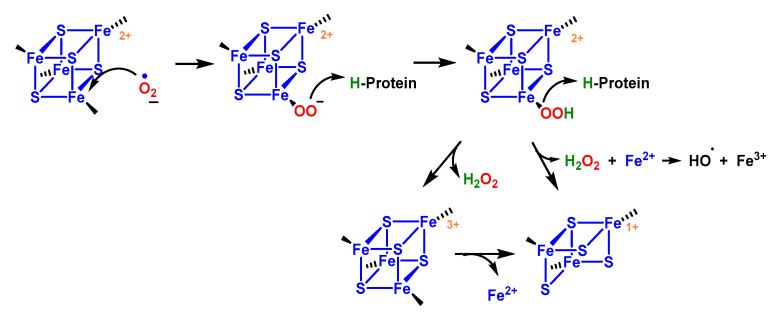
Reaction of the [4Fe-4S] cluster with O_2_^•−^. Loss of iron labile from the Fe-S centre with the consequent formation of an inactive [3Fe-4S] centre and the release of Fe^+2^.

The oxidised group is unstable, the iron atom coordinating the substrate dissociates, and the enzyme becomes inactive. Iron and hydrogen peroxide are released and may undergo other damaging reactions [114]. Consequently, inactivation of m-aconitase by superoxide can increase the formation of hydroxyl ^•^OH radicals via the Fenton reaction in mitochondria, Figure 45.

Two aconitase isoenzymes are present in mammalian cells: the mitochondrial enzyme (m-aconitase) and the bifunctional cytosolic enzyme (c-aconitase/IRP1). The assembly and disassembly of Fe-S groups is a key process, not only in regulating the enzymatic activity of mitochondrial aconitase in the citric acid cycle, but also in controlling the iron-sensing and RNA-binding activities of cytosolic aconitase (also known as iron regulatory protein IRP1). Iron deficiency can decrease aconitase protein levels and limit the assembly of Fe-S groups required for its enzymatic activities. As a result, iron deficiency can potentially affect the citric acid cycle, lipid biosynthesis, carbohydrate metabolism and many other biological pathways involving citrate.

#### 4.1.9. Conversion of Nitric Oxide to Peroxynitrite

**Figure 46:**
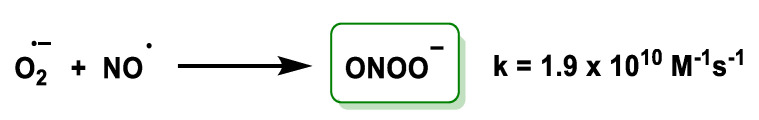
Conversion of nitric oxide to peroxynitrite and rate of reaction.

The conversion of nitric oxide to peroxynitrite is a type of reaction between two radicals. O_2_^•−^ and ^•^NO react and produce, within the mitochondrial matrix, ONOO^–^, a potent oxidant that is normally reduced by the action of mitochondrial reductants, such as NADH2, ubiquinol UQH2 and glutathione GSH. When produced in excess, because its control is lost (e.g., in ischaemia/reperfusion or inflammation), it leads to tyrosine nitration and mitochondrial dysfunction. Its cumulative effect would contribute to tissue ageing. Another radical formed extensively by both enzymatic and non-enzymatic processes is nitric oxide ^•^NO, which serves as an intra- and intercellular signalling molecule [115,116]. ^•^NO and superoxide react in a diffusion-limited manner. This reaction terminates the chain reaction initiated by superoxide, although peroxynitrite is commonly considered a harmful molecule [117], Figure 46.

This reaction has a very high rate constant and is fast enough to compete with SOD-catalysed dismutation [22,118]. Peroxynitrite is a highly reactive oxidant and is capable of undergoing a wide range of oxidative processes [119]. These include direct oxidations and secondary reactions due to nitrogen dioxide, hydroxyl and carbonate radicals [120,121].

Peroxynitrite is a short-lived and highly reactive oxidant and is therefore another reaction that confers indirect toxicity to O_2_^•−^, in particular to DNA, proteins and lipids [122]. In addition, peroxynitrite is capable of nitrating tyrosine or tryptophan residues, or oxidising methionine residues [123,124,125,126,127,128].

#### 4.1.10. Nucleophilic Substitution Reaction

**Figure 47:**
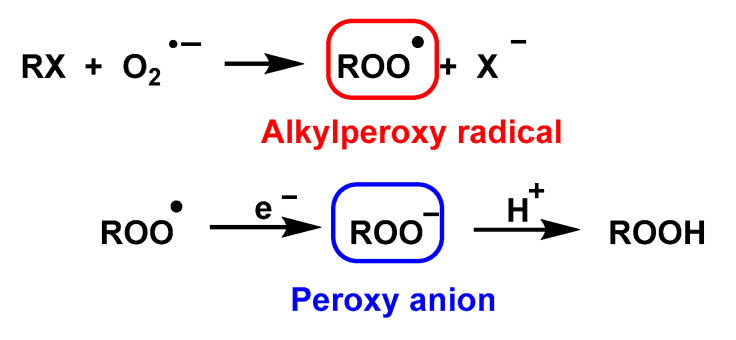
Formation of alkylperoxy radicals and peroxy anions.

O_2_^•−^ is a nucleophile in the reaction with alkyl halides and alkyl tosylates in DMSO and leads to the formation of alkylperoxy radicals and subsequently to peroxy anions via one-electron reduction [56,129], Figure 47.

**Figure 48:**
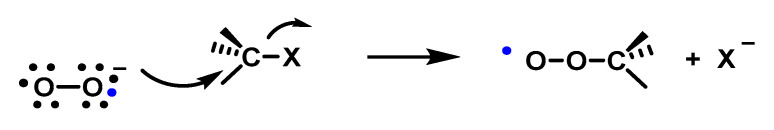
Formation of the C-O bond by a mechanism involving a displacement of SN2 on carbon.

The high stereoselectivity observed in this reaction is inconsistent with the intermediation of free alkyl radicals. Furthermore, it has been observed that the structure of the alkyl group attached to the halogen, the nature of the leaving group, and the polarity of the solvent exert influence on the course of the reaction in the carbon–oxygen bond formation step that is consistent with a mechanism involving an SN2 displacement on the carbon; therefore, it follows that the initial reaction of the superoxide with an alkyl halide produces an alkylperoxy radical [130], Figure 48.

#### 4.1.11. Reactions of Superoxide with Amino Acids

**Figure 49:**
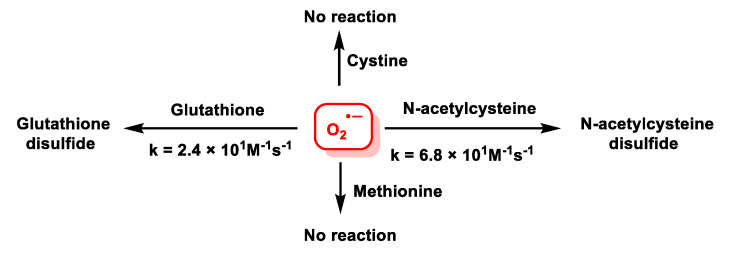
Rate constants for the reaction of N-acetylcysteine and glutathione with O_2_^•−^.

Second order rate constant values for HO_2_^•^ reactions for aliphatic amino acids are reported in the literature in the range of 1 × 10 M^−1^s^−1^ and a value of about 6 × 10^2^ M^−1^s^−1^ for Cys, these values are even lower for O_2_^•−^ reactions of these amino acids, with values ranging from 1.0 × 10^−1^ M^−1^s^−1^ to about 2 × 10 M^−1^s^−1^ [131]. The difference in reactivity of the two superoxide species may be due to the fact that HO_2_^•^ acts as a weak oxidising agent, while O_2_^•−^ behaves as a weak oxidising and reducing agent, and these values may also depend on whether the amino acids are protonated or not. Cystine and Met do not react with superoxide, but N-acetylcysteine and glutathione GSH do [132,133,134], Figure 49.

**Figure 50:**
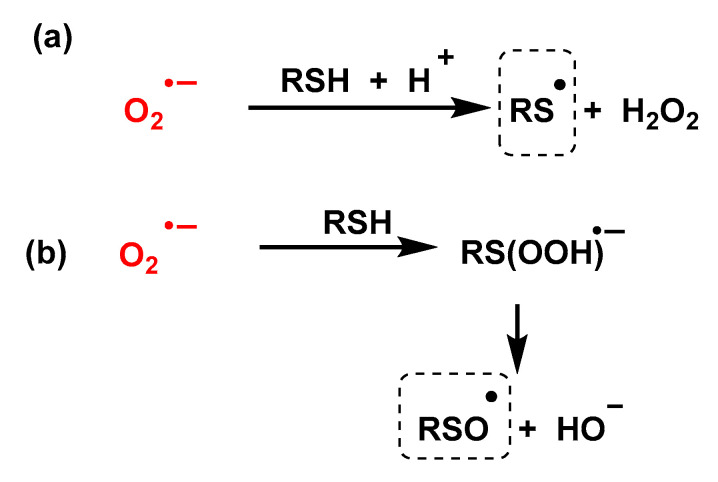
Two pathways for the reaction of O_2_^•−^ with thiols.

Despite the large body of experimental work dealing with the oxidation of thiols by superoxide, the mechanism of this reaction remains controversial, Figure 50. According to some authors [135,136,137], the superoxide radical reacts with RSH thiols by abstraction of the hydrogen atom giving the thiyl radical and hydrogen peroxide; other authors consider that the first step of the reaction is the formation of a three-electron-bond radical, which breaks into a sulfinyl radical and a hydroxide anion [132,133,134].

In both reactions it is assumed that the intermediate RS or RSO will further interact with the thiolate to give the final disulphide RSSR.

#### 4.1.12. Radical–Radical Reactions of Superoxide

**Figure 51:**
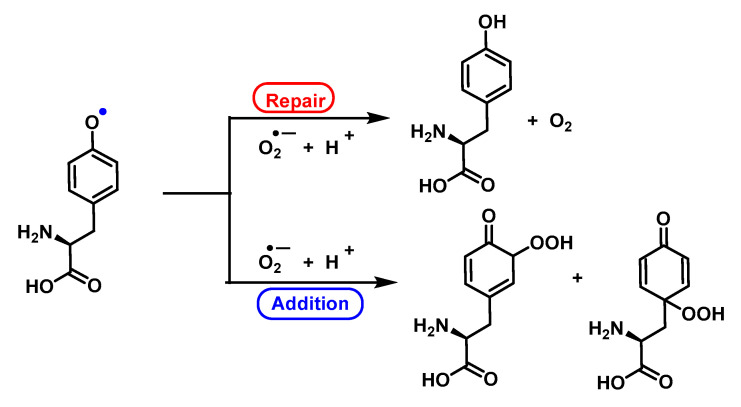
The reaction of tyrosyl radicals with O_2_^•−^ provides tyrosine and tyrosine hydroperoxides. Electron transfer repairs the Tyr radical and addition results in the formation of Tyr-hydroperoxide.

**Figure 52:**
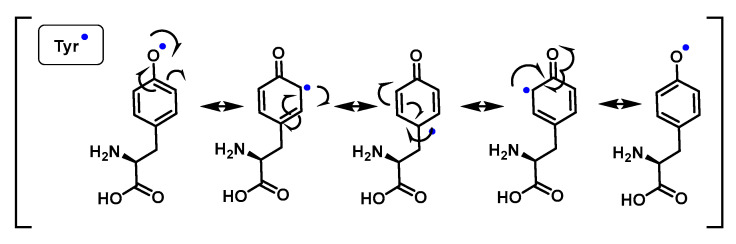
Resonant shapes of the Tyr^•^.

Phenoxyl radicals are produced by one-electron oxidation of phenols. The most common phenol in biological systems is tyrosine, either in its free form or in peptides and proteins. The phenoxyl radicals in Tyr initially react with superoxide by addition, and the intermediate formed either releases oxygen to regenerate the parent compound or is converted to a hydroperoxide. The hydroperoxide, due to the resonant forms of Tyr, can be formed in the ortho or para position with respect to the phenoxyl radical [138,139,140], Figure 51 and Figure 52.

**Figure 53:**
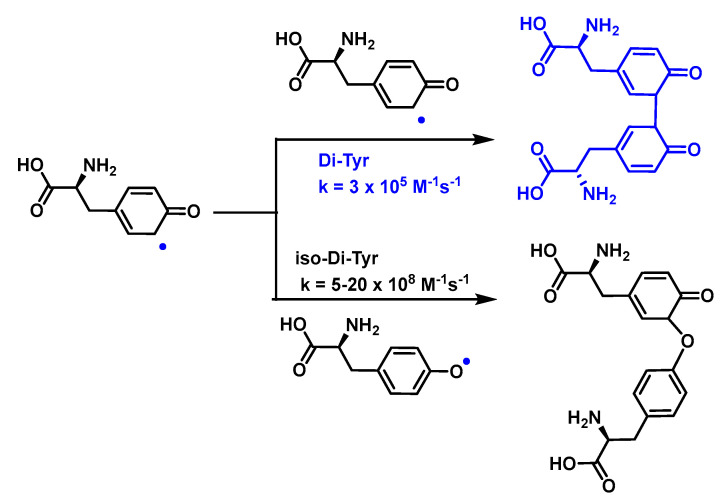
Formation and reactions of Tyr phenoxyl radicals Tyr^•^. Tyr^•^ self-react to produce di-Tyr (o,o′-di-Tyr, blue; iso-di-Tyr, black).

As a consequence of electron delocalisation by the benzene ring, the Tyr^•^ undergo rapid dimerization reactions to give Tyr-Tyr cross-linked species producing isomers with different cross-linked bonds, C-C cross-linked and C-O cross-linked species, Figure 53.

Reactions of superoxide with tyrosyl radicals on the original amino acid or on small peptides are fast enough to compete with both superoxide dismutation and tyrosyl radical combination to form dityrosine derivatives. The production of di-Tyr crosslinks is limited by these alternative reactions, such as the short-lived peroxide formation reaction. For tyrosyl radicals, the reaction with superoxide to form the addition product (tyrosine hydroperoxide) is three times faster than dimerization [139,141].

**Figure 54:**
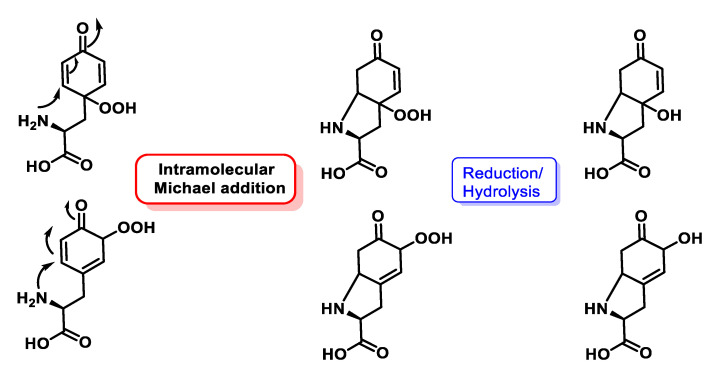
When tyrosine is N-terminal, the major products are hydroperoxides that have cyclized via intramolecular Michael addition of the terminal amine.

Tyrosine and aminophenols are anomalous in that they undergo many more additions, the amino group attaches to the b-position of the unsaturated a,b.ketone to give a heterocyclic product. When tyrosine is N-terminal, the main products are hydroperoxides that have been cyclised via 1,4-conjugated addition (intramolecular Michael addition) of the terminal amine, Figure 54. When tyrosine is not N-terminal, electron transfer from O to the peptide radical prevails. Superoxide and tyrosyl radicals are among the most frequent radicals generated biologically during oxidative stress. The reaction between the two is very favourable. O_2_^•−^ can react rapidly with low molecular mass Tyr- radicals, with a rate constant of ~1.5 × 10^9^ M^−1^ s^−1^, which is about three times faster than dimerization, Figure 54.

**Figure 55:**
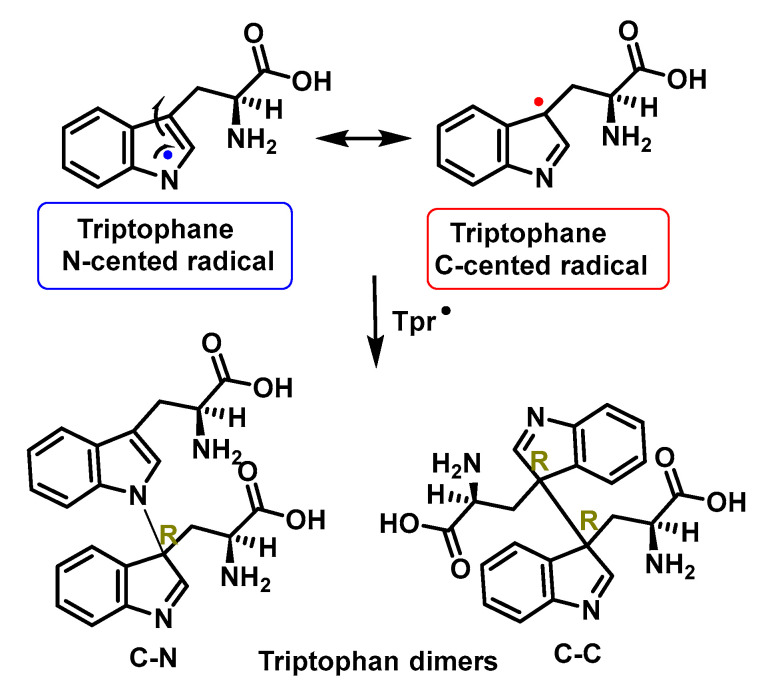
Proposed structures of isomeric Trp-Trp crosslinks. As a result of delocalization of electron density around the indole ring, Trp^•^ can undergo dimerization reactions that produce isomers with different C-C and C-N bonds. The structures indicate R stereochemistry around the formed bonds.

An indole radical (tryptophanyl radical Trp-) is readily formed on the indole ring of tryptophan Trp. These radicals undergo multiple reactions, including ring opening and dimerization. As a consequence of electron delocalisation on the indole ring, Trp^•^ radicals undergo rapid dimerization reactions to give Trp-Trp cross-linked species that produce isomers with different cross-linkages, C-C cross-linked and C-C cross-linked species. C-N [142,143], Figure 55.

Trp^•^ can undergo rapid dimerization to form a series of isomeric cross-linked Trp-Trp species. These reactions have second-order rate constants in the range of k = 2–6 × 10^8^ M^−1^ s^−1^ at pH 7.4 and k = 7.3 × 108 M^−1^ s^−1^ at pH 10. These high values suggest that dimerization can compete with other Trp- reactions in complex systems. Trp^•^ dimerization takes place under conditions where O_2_^•−^ is absent or present in low concentrations [144].

**Figure 56:**
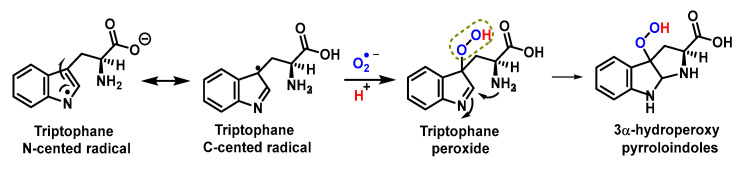
Trp^•^ reacts with O_2_^•−^, the major products being hydroperoxides.

The reaction of Trp^•^ radical with O_2_^•−^ gives a hydroperoxide. Trp^•^ reacts very rapidly with O_2_^•−^ with second order rate constants, k, in the range 0.7–2.2 × 10^9^ M^−1^ s^−1^, the main initial products being hydroperoxides, in almost quantitative yields [145,146,147,148,149,150,151], Figure 56.

**Figure 57:**
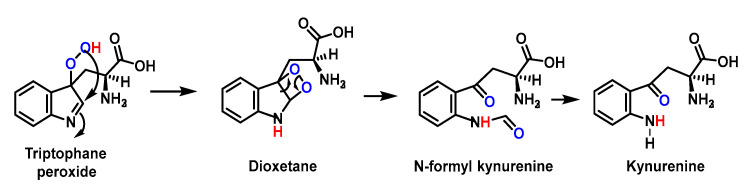
The formation and subsequent decomposition of dioxetanes gives as the main product N-formylkynurenine NFK which is hydrolysed to kynurenine Kyn.

Subsequent decomposition of these species gives rise to N-formylquinurenine, quinurenine, alcohols and diols. These data indicate that the O_2_^•−^ with Trp^•^ reaction should be considered as an important pathway for the degradation of Trp in peptides and proteins subjected to oxidative damage, Figure 57.

#### 4.1.13. Proton–Radical Transfer

Because the pKa of the conjugate acid of O_2_^•−^ is 4.8, O_2_^•−^ is considered a weak base. However, proton and radical transfer pathways have been proposed to demonstrate the antioxidant property of phenols and polyphenols against O_2_^•−^. Results obtained by cyclic voltammetry show that proton transfer and radical transfer pathways are present for both monophenols and polyphenols, and the relative contributions of the two pathways depend on the structure of the phenol. Polyphenols containing an o-diphenol ring (as flavonoids) have the highest reactivities.

**Figure 58:**
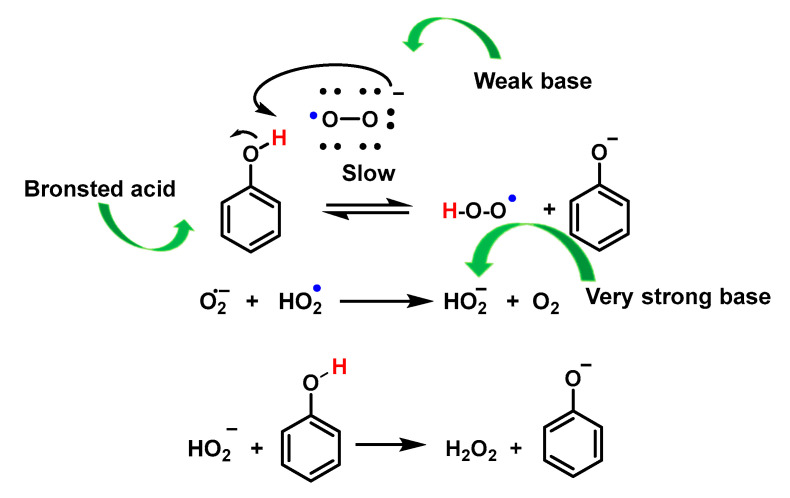
Reaction of phenol with superoxide anion.

Proton transfer is the main mechanism for the reaction between monophenols and O_2_^•−^ in aprotic solvents, such as DMF or DMSO. The mechanism involves a first proton transfer between O_2_^•−^ acting as a weak base and the phenolic compound PhOH acting as a Bronsted acid according to Figure, in which the formation of phenoxide PhO^–^ and HO_2_^•^, although thermodynamically unfavourable, can be completed by the electron transfer reaction between HO_2_^•^ and O_2_^•−^ to form HO_2_^•^ (a very strong base) and O_2_. In this, the former can extract more protons from the phenol to form the phenoxide PhO^–^ according to Figure, in which the former can extract more protons from the phenol to form the phenoxide PhO^–^ according to Figure 58.

**Figure 59:**
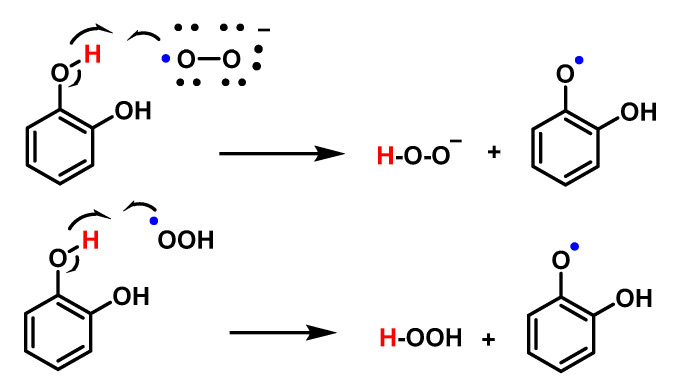
The reaction of QH2 with HO_2_^•^ and O_2_^•−^ provides radical semiquinone.

Polyphenols, however, undergo a radical (or H-atom) transfer reaction with O_2_^•−^ to form the phenoxyl radical PhO^–^ and HO_2_^•^. Similar to monophenols, HO_2_^•^ can also extract protons from PhOH to form phenoxide PhO^–^, Figure 59.

The reactivity of HO_2_^•^ with QH2 involves an H-atom transfer reaction to form a semiquinone radical and H_2_O_2_ with a rate constant of 4.7 × 10^4^ M^−1^ s^−1^ for 1,2-dihydroquinone.

**Figure 60:**
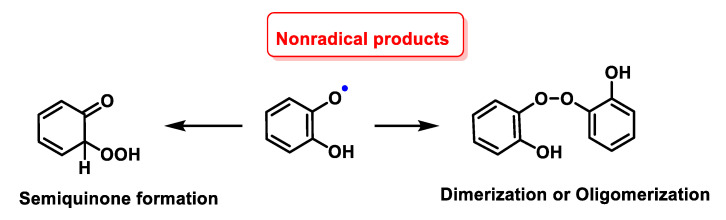
PhO^•^ forms non-radical products by dimerization, oligomerisation or formation of semiquinone.

PhO^–^ was shown to form non-radical products via dimerization or oligomerisation, or semiquinone formation. This difference in the pathway between the decomposition of monophenols and polyphenols with O_2_^•−^ may be due to the stabilisation of the radical in polyphenols through resonance, as shown by the higher reactivity of polyphenols containing o-diphenol rings with O_2_^•−^, Figure 60.

## 5. Detection of Superoxide Anion

There are a large number of published methods for superoxide detection, with their advantages and disadvantages. The simplest are assays that measure superoxide in free solution or when it is released from cells. What they all have in common is that they employ what has been termed an indicating scavenger, which is a molecule that reacts with superoxide to produce a detectable product. Commonly used chemiluminescent scavengers are lucigenin, luminol or cytochrome C.

### 5.1. Detection of Superoxide by Cytochrome C

**Figure 61:**
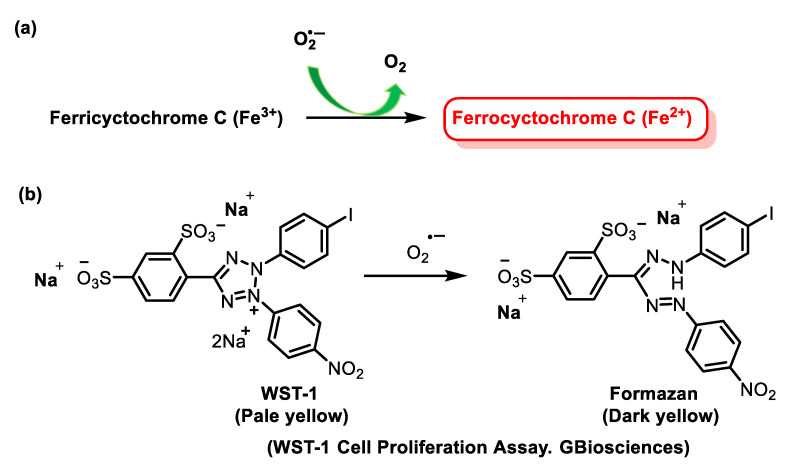
(**a**) Detection of O_2_^•−^ by reduction of a cytochrome C system, (**b**) reduction of WST-1 in a formazan salt.

The neutrophil respiratory burst is usually measured spectrophotometrically: (a) following the reduction of ferricytochrome C Fe^3+^ which is reduced by superoxide, producing oxygen and bright-red ferrocytochrome C Fe^2+^ with a detectable spectrophotometric absorbance at 550 nm [152,153]; and (b) histologically using the tetrazolium salt, nitro blue tetrazolium, which is reduced intracellularly to an insoluble formazan. In both assays, the reduction is mediated by a superoxide generated via NADPH oxidase. Because ferricytochrome c has a high molecular mass and a high background absorbance at 550 nm, the assay lacks sensitivity and is not ideal for measurement in microplates. To overcome this limitation, the cell-impermeable sulphonated tetrazolium salt, WST-1, which exhibits a very low background absorbance and is efficiently reduced by superoxide to a stable, water-soluble formazan with high molar absorptivity, is used [154,155], Figure 61.

Cytochrome C is a traditional and accurate method for detecting large amounts of superoxide. However, it should be noted that electrons donated by enzymes or other molecules can also reduce ferricytochrome C, resulting in an absorbance that is not specific to superoxide.

### 5.2. Fluorescent Probes

The determination of oxidative stress in cells is performed by assays based on fluorescent tests. These techniques are based on the addition of a non-fluorescent compound (fluorogenic substrate), which is rapidly oxidised upon reaction with oxidising species, transforming it into a fluorescent compound and whose emission is detected by the flow cytometer or fluorometer. Most fluorescent probes developed to date for in vitro and in vivo imaging are designed to be activated in the presence of the analyte of interest. Activation usually results in an increase in fluorescence or a change in emission wavelength. The advantage of these probes is the easy visualisation of intracellular dynamics and high-resolution localisation of biomolecules of interest. Most of the widely used probes that are commercially available are prefluorescent aromatic molecules that oxidise in the presence of ROS to a fluorescent product. Many of the probes developed in recent years are compounds containing a masked fluorophore that is released by the attack of the oxidant on the masking group.

The above assays are not suitable for intracellular or tissue analysis of superoxide, and fluorescent or chemiluminescent probes have been developed to achieve this goal. Most of these probes react through multistep mechanisms that can be influenced by a number of variables. The most commonly used probes for superoxide are dihydroethidium and its analogues. For these assays to show true values, they must be coupled with a chromatography system using fluorogenic probes to detect 2-hydroxyethidium, which is the species formed by superoxide.

Other detectors, such as lucigenin, also react with other ROS, such as superoxide, but also generate superoxide as part of their mechanism, so they are not suitable for determining the amount of O_2_^•−^ generated. Some of the chemiluminescent probes show improved sensitivity [156] but there are still problems with interference [157].

**Figure 62:**
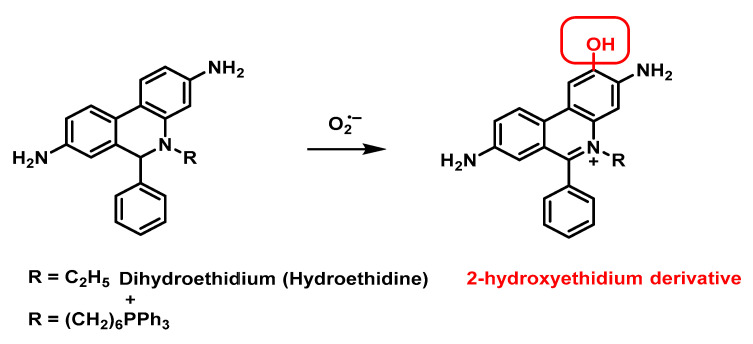
Fluorescent response mechanism of the dihydroethidium (hydroethidine) probe and derivatives to O_2_^•−^.

Hydroethidine has been widely used to detect the intracellular superoxide radical, in the reaction between these two species 2-hydroxyethidium is generated. The production of superoxide radicals in amounts as low as 1.5 pmol in 5 mg of biological samples can be quantified fluorometrically with this probe [158], Figure 62.

Superoxide dihydroethidium indicator, also called hydroethidine, fluoresces blue in the cytosol until it oxidises, where it intercalates into the cell’s DNA and stains its nucleus a bright fluorescent red.

Other variations of the hydroethidine probe exist, for example one with a triphenylphosphonium residue on the nitrogen atom. It is suggested that these two probes fluoresce through superoxide-induced oxidation of the probes to result in the formation of the corresponding 2-OH-ethidium products, which can be excited at both 396 and 510 nm. Therefore, by controlling the fluorescence emission at these excitation wavelengths, superoxide-induced oxidation can be differentiated from other cellular oxidative processes.

**Figure 63:**
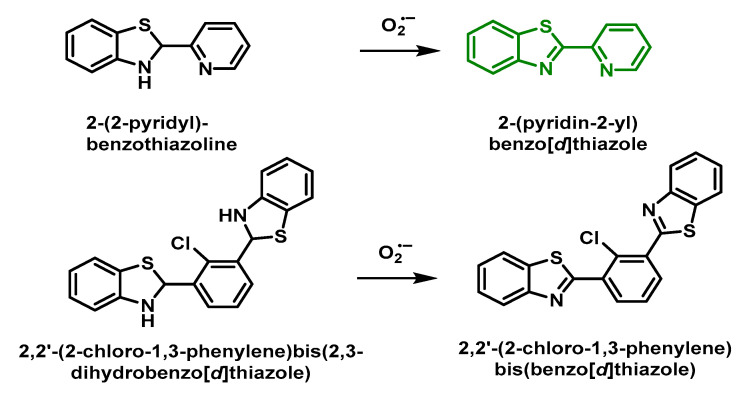
Response mechanism of the 2-(2-pyridyl)-benzothiazoline and 2,2′-(2-chloro-1,3-phenylene) bis(2,3-dihydrobenzo [d] thiazole) probe to O_2_^•−^.

Tang’s group described two O_2_^•−^-sensitive probes derived from benzothiazoles, 2-(2-pyridyl)-benzothiazoline and 2,2′-(2-chloro-1,3-phenylene) bis (2,3-dihydrobenzo [d] thiazole). Oxidation by O_2_^•−^ leads to a strongly fluorescent product [159], Figure 63.

The same research group also developed the fast-response fluorescent probe 2,2′-(2-chloro-1,3-phenylene) bis (2,3-dihydrobenzo [d] thiazole) which operates by a similar detection mechanism in the presence of O_2_^•−^ and also shows a high selectivity towards O O_2_^•−^ in contrast to other ROS [160].

**Figure 64:**
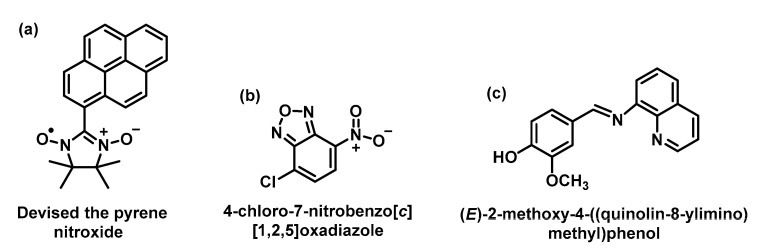
Structure of pyrene-derived probes nitroxide (**a**), oxadiazole (**b**), and imine derivative (**c**).

Medvedeva et al., 2004, devised the pyrene-nitroxide-based probe to control the amount of superoxide generated by using a xanthine/xanthine oxidase system, Figure 64. This probe is not fluorescent and is not specific to O_2_^•−^ because it also responds to the hydroxyl radical and some antioxidants, indicating its poor selectivity [161].

The 4-chloro-7-nitrobenzo [c] (1,2,5) oxadiazole probe is commercially available and is used for the rapid detection and quantification of the superoxide radical generated by the xanthine/xanthine oxidase system [162]. The drawback of this probe is that solutions containing 66.7% acetonitrile are required for the detection system, which limits biological applications.

The imine (E)-2-methoxy-4-((quinolin-8-ylimino) methyl) phenol formed by the reaction of vanillin with 8-aminoquinoline was also used as a fluorescent probe for the detection of O_2_^•−^ [163]. In the presence of O_2_^•−^ this probe is oxidised to form a quinoid-containing product, leading to a decrease in fluorescence intensity.

**Figure 65:**
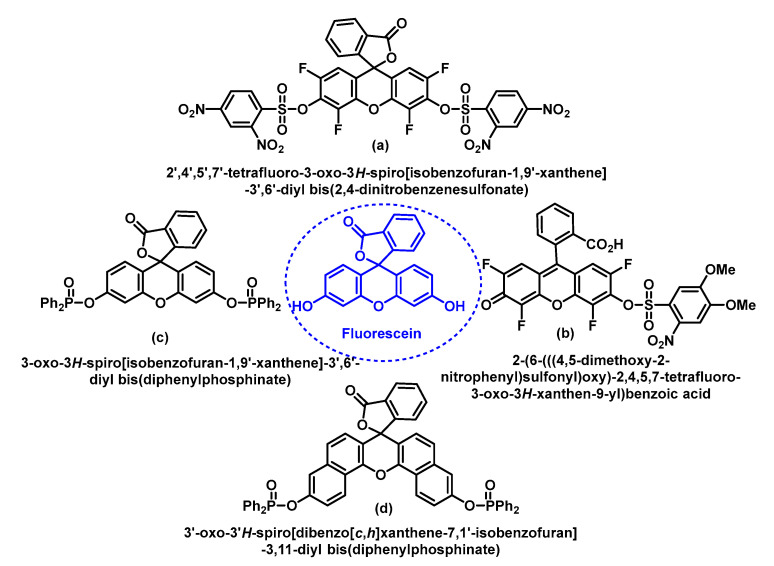
Four fluorescein-derived probes used for O_2_^•−^ detection.

Shown in the figure are the four fluorescein-derived probes used for O_2_ detection—(a) and (b) were developed in Maeda’s group and (c) and (d) in Tang’s group, Figure 65.

**Figure 66:**
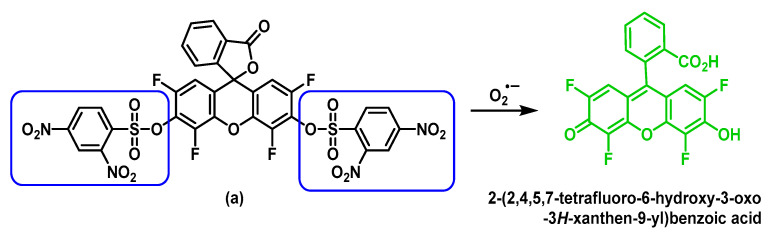
Mechanism of fluorescent response of probe (a) to O_2_^•−^.

Other highly specific fluorescent probes (a) and (b) for the detection of O_2_^•−^ were developed by Maeda’s group based on a non-redox mechanism [164]. As shown in Figure 66, after treatment with O_2_^•−^, the benzenesulphonate groups are removed and the product formed produces a large increase in fluorescence.

Additionally, based on a strategy of deprotection of the benzenesulfonyl group, Maeda et al., 2007, demonstrated that probe (b) serves as an optimal sensor for the detection of O_2_^•−^ in aqueous solutions and pH 7.4, the detection limit of this probe for O_2_^•−^ is 10 times higher than that of probe (a). In addition, probe (b) shows a high selectivity towards O_2_^•−^ over glutathione and other ROS, such as hydrogen peroxide, hypochlorite, tert-butyl peroxide, singlet oxygen, ^•^NO and peroxynitrite [165].

**Figure 67:**
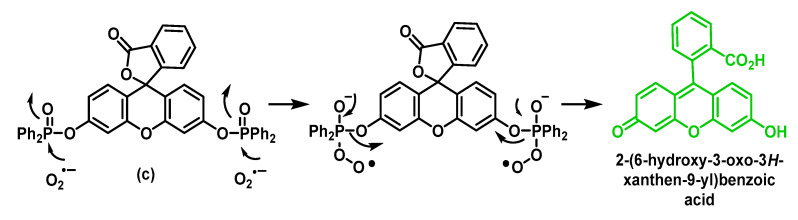
Mechanism of fluorescent response of probe (c) to O_2_^•−^.

The fluorescent probes (c) and (d) were designed by Tang and co-workers, and their functions are based on the nucleophilic property of O_2_^•−^ which is involved in the deprotection of the diphenylphosphinate residues attached to the fluorescein probe [166] (c), Figure 67, or the naphthofluorescein probe [167] (d). In aqueous solution pH 7.4, both probes show high sensitivity. In addition, they have excellent selectivity towards O_2_^•−^ over other ROS/RNS.

### 5.3. Chemiluminescent Probes

**Figure 68:**
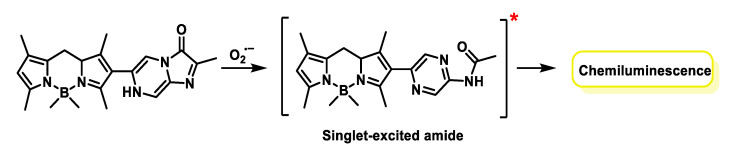
Mechanism of chemiluminescence response of probe BODIPY.

Suzuki and co-workers designed the chemiluminescent probe derived from 4-4-diflouro-4-bora-3a,4a-diaza-s-indacenes known as BODIPYs. BODIPYs (short for borodipyrromethene) are fluorescent molecules (or fluorochromes) that have advantageous features, such as thermal and photochemical stability, high solubility and chemical robustness, among others for O_2_^•−^ detection. The response of this probe to the superoxide radical is triggered by the formation of a dioxetanone that decomposes to generate a singlet excited amide, which decays to its ground state with simultaneous emission of yellow-green luminescence (545 nm). Highly sensitive detection of O_2_^•−^ generated from PMA-stimulated HL-60 cells was demonstrated using the probe shown in Figure 68.

**Figure 69:**
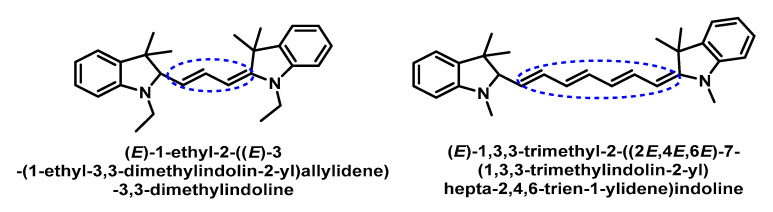
Structure of probes derived from hydrocyanines.

Murthy’s group found that hydrocyanines could be used to detect the superoxide [168]. The two probes studied by this group are shown in Figure 69.

**Figure 70:**
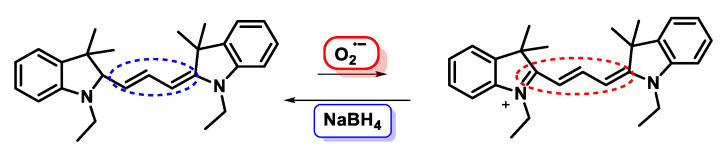
The oxidation–reduction reaction of hydrocyanines and cyanines.

Hydrocyanines are generated from cyanine dyes by reduction with NaBH_4_, Figure 70.

Hydrocyanines are weakly fluorescent because they contain an interrupted π-conjugated system. However, oxidation of these molecules with the superoxide radical enhances their fluorescence through the generation of extended π-conjugation products. The hydrocyanine-derived probes show a high selectivity towards oxidising radicals.

## 6. Can Superoxide Anion Repair Oxidative Damage?

Muñoz-Rugeles et al., 2018, have shown that superoxide is able to repair oxidised DNA by transferring an electron to the guanosyl radical of single-stranded DNA. They have considered acid–base equilibria and explored the influence of pH on the main reaction mechanism. Superoxide reactivity is not necessarily detrimental to biomolecules but can also help combat oxidative damage. Unfortunately, this type of involvement in chemical processes has not yet been studied in depth [169].

## 7. Superoxide Anion in the Antimicrobial Innate Immunity

O_2_^•−^ is involved in many aspects of the immune response to pathogens, as it can damage biomolecules by oxidizing iron and sulphur groups in a variety of enzymes, leading to metabolic defects and iron release. O_2_^•−^ is essential for the elimination of pathogens by phagocytic cells, as seen in patients suffering from chronic granulomatous disease CGD, an inherited NADPH oxidase disorder characterised by recurrent and severe bacterial and fungal infections [170]. Alongside the ability of O_2_^•−^ to induce lysis of bacteria in the phagosome through oxidative damage to bacterial biomolecules, defence against pathogens is also triggered by various non-oxidative means, such as autophagy, receptor signalling, extracellular trap formation and instruction of lymphocyte responses [171].

In the phagocytosis area, O_2_^•−^ production by the phagocyte NOX has been associated with pathogen killing for the last decades. In contrast, at much lower concentrations, O_2_^•−^ and ROS are necessary for cell signalling. O_2_^•−^ produced by NOX acts as a signalling molecule by modifying the redox state of proteins or lipids, and one of its possible targets is even NOX itself. Superoxide and H_2_O_2_ added to NOX subunits have been shown to decrease O_2_^•−^ production, but only when added prior to subunit assembly [172]. Additionally, O_2_^•−^ derived from different NOx influences distinct downstream signalling pathways, which may be the reason for the co-expression of more than one isoform of NOX in specific cell types [173].

Phagocytes can release O_2_^•−^ both into the phagosome and the extracellular space due to expression of NOX2 on both the phagosome and the plasma membrane [174]. The activation of the mammalian phagocyte NOX2 is tightly regulated and predominantly depends on the engagement of surface receptors by dedicated ligands. Some cell surface receptors (Toll-like receptors (TLR), G-protein-coupled receptors (GPCR) and TNF receptors (TNFR)), can prime the NOX2 activation [175]. Stimulation of other receptors, including Fc and integrin, result in direct activation of NOX2.

As NOx is assembled, electrons pass from cytosolic NADPH to FAD and membrane-integrated heme groups and begin the reduction of molecular oxygen O_2_ to O_2_^•−^. Superoxide O_2_^•−^ is a highly reactive “non-diffusible” specie that is enzymatically transformed to hydrogen peroxide. In granulocytes, H_2_O_2_ is quickly converted into hypochlorous acid HOCl [176] by the action of the enzyme myeloperoxidase (MPO) contained within them. HOCl has superior bactericidal characteristics to superoxide [177].

The role of superoxide in phagocytes differs depending on NOX2 expression and activity. After activation, neutrophils produce more O_2_^•−^ compared to monocytes and macrophages [178]. Dendritic cells (DC) express little NOX2 and consequently have a lower O_2_^•−^ and ROS production after activation [179]. In neutrophils, H_2_O_2_ generation in phagosomes activates the enzyme myeloperoxidase, catalysing the production of HOCl, which is oxidant and antimicrobial, and contributes greatly to the lysis of microbes [178]. After infection, excessive neutrophil activity causes tissue damage, so their deactivation or cell death is physiologically important and tightly regulated. This process is called pathogen-induced cell death and is also dependent on NOx activity [180]. Mononuclear phagocytes (primarily monocytes and macrophages) do not express MPO and thus contain more H_2_O_2_ in their phagosomes.

## 8. Macrophages, Neutrophils and Superoxide Anion

### 8.1. Macrophages

Macrophages, discovered by Ilya Metchnikoff in the late 19th century, were long considered an important part of the effector cells of the immune system. Metchnikoff won the Nobel Prize in 1908 for his description of phagocytosis and went so far as to propose that the key to immunity was to “stimulate phagocytes” [181]. Irrespective of their role in the immune system, macrophages clear about 2 × 10^11^ erythrocytes daily and are also involved in the removal of cellular debris generated during tissue remodelling, thereby eliminating cells that have undergone apoptosis. These clearance processes are a vital metabolic contribution, without which the host would not survive [182].

Macrophages (from Greek: large eaters, makros + phagein), are the first immune cells to encounter invading pathogens, and their goal is to engulf microbes, dead cells, foreign substances, cancer cells and cellular debris by phagocytosis [183]. These phagocytes are found essentially in all tissues, and historically, they have been given various names, as histiocytes, Kupffer cells, alveolar macrophages, microglia and others, all of which are part of the mononuclear phagocytic system [184]. They play a key role in nonspecific defence (also called as innate immunity) and help specific defence mechanisms (adaptive immunity) by recruiting other immune cells (lymphocytes) [185]. If a person has dysfunctional macrophages, this causes diseases, such as chronic granulomatous disease, which leads to frequent infections, as this first line of defence against infection is not available [186]. Human macrophages are about 21 μm in diameter and are produced by the differentiation of monocytes in tissues [187].

Macrophage activation is a response to a wide range of stimuli, including the nature of microbial agents, damaged cells, activated lymphocytes and inflammatory cells [188]. Classically activated macrophages require a priming signal in the form of interferon IFN-γ [189]. Priming of macrophages with IFN-γ reprograms cellular responses to other cytokines, such as type I IFNs and IL-10. Alternatively activated macrophages do not require any priming, because interleukins IL-4 and/or IL-13 can act as enough stimuli [190,191]. Alternatively activated macrophages change their morphology and chemical secretion pattern as a result [192].

The bioplasticity of macrophages is one of their main characteristics, resulting in extreme heterogeneity under physiological but also pathological conditions. Macrophages can be classified into two distinct subsets: M1, or classically activated, and M2, or alternatively activated [193]. This plasticity induces a phenomenon called polarisation, which regulates the functionality of macrophages and the role they will play in the tissues that host them [194].

M1 macrophages are pro-inflammatory and polarized by lipopolysaccharides (LPS) and/or cytokines, such as IFN-γ and the granulocyte-macrophage colony-stimulating factor GM-CSF, and produce pro-inflammatory cytokines, such as interleukin-1β IL-1β, IL-6, IL-12, IL-23 and TNF-α [194]. They secrete high levels of TNF-α, IL-12 and IL-23 cytokines. M1 macrophages produce intracellular nitric oxide ^•^NO by inducible nitric oxide synthase iNOS and superoxide anion O_2_^•−^ and subsequent reactive species, which are cytotoxic to microbial agents and fight infection [195].

M2 macrophages are polarized and activated upon exposure to certain cytokines such as IL-4, IL-10 or IL-13, subsequently producing polyamines and/or proline, which are involved in cell proliferation and collagen production [196]. These M2 macrophages are associated with wound healing and tissue repair. The secretion of the inflammation-inhibiting cytokine IL-10 is the hallmark of M2 macrophages, arginase-1 and more organic compounds [197]. This phenotype can be further subdivided into M2a, M2b and M2c, based on their specific functions. All three subtypes have anti-inflammatory properties, but M2a and M2b macrophages are considered immunoregulatory and are known to mediate the Th-2 response. M2c cells are immunosuppressive and are involved in extracellular matrix ECM remodelling. M2a and M2c secrete growth factors that promote the formation of new blood vessels (angiogenesis) and tissue regeneration [198].

Finally, it is increasingly clear that macrophages are involved in the progression of pathological conditions, including cancer, cardiovascular disease, obesity and wound healing [196]. In cancer, M1 macrophages have anti-tumour functions, while M2 macrophages support angiogenesis, invasion and metastasis of neoplastic cells. Most tumour-associated macrophages (TAM) adopt an M2-type phenotype, and their presence correlates with a poor prognosis [199,200].

Alongside O_2_^•−^ production, macrophages employ several direct antimicrobial mechanisms in the phagosome, such as reactive nitrogen species RNS, as well as delivery of cathepsins and other hydrolases [201,202,203,204]. Indirect mechanisms involve inflammasome activation and the secretion of cytokines and chemokines, which aim to organize subsequent innate and adaptive immune responses, as well as MHC-dependent presentation of pathogen-derived antigens [205].

When macrophages initiate the bacterial recognition process, O_2_^•−^ production starts in different cellular compartments [201]. One of the most important goals is the lysis of phagocytosed bacteria by the oxidative burst generated by the NADPH oxidase NOX2. The oxidative burst is the rapid release of O_2_^2-^ from various cell types, especially macrophages and neutrophils, and it requires a 10- to 20-fold increase in oxygen consumption by NOX activity. The oxidative burst in phagocytes is usually associated with killing bacteria, but in the case of alveolar macrophages, they usually produce lower levels of ROS than neutrophils and may need to be activated to exert their bactericidal properties. Instead, their transient oxidative burst regulates the inflammatory response by triggering the synthesis of cytokines for redox signalling, resulting in an influx of activated neutrophils and macrophages.

### 8.2. Neutrophils

Neutrophils are a main cellular component of the innate immunity and play a dual role because they provide a rapid and non-specific response to the infectious progression and mediate between the innate and the adaptive immune systems. Neutrophils are short-lived granulocytes derived from pluripotent hematopoietic stem cells from bone marrow [206]. Most haematopoiesis is related to granulopoiesis, and almost 60% of bone marrow leukocytes are granulocyte precursors [207].

Neutrophils are the main leukocytes circulating in the blood, and the first innate immune cells to be recruited to a focus of infection. Here, neutrophils have several defence strategies at their disposal, such as the production of superoxide O_2_^•−^, the release of antimicrobial factors and the formation of neutrophil extracellular traps (NET) [208].

There are two main granule populations in mature neutrophils: (i) azurophils are the first to develop during granulopoiesis, they contain the enzyme myeloperoxidase (MPO) and other proteolytic enzymes (cathepsins, proteinase-3, elastase), antimicrobial defensins and bactericidal proteins [209]; and (ii) a specific type of granules (peroxidase negative) that mature during differentiation contain membrane proteins, such as lactoferrin and collagenase, and are receptors for chemotactic peptides, cytokines, opsonins and adhesion proteins [210]. Mature neutrophils are released into the bloodstream, where they circulate for 10–24 h before migrating to tissues, where they remain for the next 1–2 days before undergoing apoptosis and being cleared by macrophages [211]. Neutrophils released into the systemic circulation constitute most of the circulating leukocyte cell population. Under normal conditions, the number of mature neutrophils is almost constant, but during an infectious process their population can increase up to 10-fold [212].

Some circulating neutrophils move through the walls of postcapillary veins through transient interactions with endothelial cells [213]. Their role is to look for signs of tissue damage, inflammation or the invading microorganisms themselves, as well as the presence of chemotactic or chemoattractant signals derived from the host and/or pathogen [214]. In the presence of pathogens, different host cells (such as monocytes and macrophages) secrete inflammatory and neutrophil chemoattractant mediators (such as the leukotriene LTB4 involved in inflammation, the interleukin IL-8, and the chemokine CXCL6, a small cytokine belonging to the CXC chemokine family, also known as granulocyte chemotactic protein 2 GCP-2) which bind to specific receptors on the neutrophil surface [215]. These signals direct neutrophils from the intravascular space to the site of infection in tissues.

Another important fact is neutrophil priming described in the early 1980s. Its classical definition is the ability of a primary agonist, under stimulatory concentrations, to enhance superoxide production in response to a secondary stimulus [216]. Priming occurs at almost all levels of neutrophil function: adhesion, phagocytosis, cytokine secretion, leukotriene synthesis and degranulation. Priming is induced by cytokines, chemokines, growth factors, lipid-derived signalling molecules and physical cell–cell contact and adhesion [217]. Following recognition of chemotactic signals and/or priming of neutrophils, neutrophils leave the peripheral circulation by transmigration through the endothelial wall, a process termed extravasation [209].

At the site of infection, neutrophils bind and ingest the invading micro-organisms by phagocytosis. Two main mechanisms account for the microbicidal properties of neutrophils:

(i) The production of O_2_^•−^. Neutrophils possess the enzyme NOX which, when activated, produces superoxide anion, with strong antimicrobial properties. Superoxide can be released outside the cell, or inside the cell (in the phagosome);
(ii) The coordinated release of proteolytic and antimicrobial granule content. The release of the contents of the primary and secondary granules has important antimicrobial significance. The granules contain MPO, lactoferrin, lysosomes and NGAL [218]. The enzyme myeloperoxidase MPO forms hypochlorous acid HOCl [176] after reaction of chloride anion with hydrogen peroxide. HOCl oxidises tyrosine residues to form the tyrosyl radical [128].

Because of their common origin, neutrophils and macrophages have common functions (phagocytosis) and similar kinetic performance during infections [218]. Neutrophils can influence macrophage differentiation into pro- or anti-inflammatory subtypes [218]. Interferon-γ released by activated neutrophils induces macrophage activation [219]. Neutrophils release MPO, which is taken up by residential macrophages expressing macrophage mannose receptors (MMR). The interaction between MPO and MMRs leads to the release of ROS (reactive oxygen species) and proinflammatory cytokines (IL-6, IL-8, TNF-α, IL-1, GM-CSF) by macrophages. The release of TNF-α, IL-1β, G-CSF and GM-CSF at the site of macrophage infection increases the survival of recruited neutrophils from 6–12 h to 24–48 h [220]. The cytokine IL-17 is an element of the innate immune system released by CD4+ Th17, NK cells and neutrophils. IL-17 acts on neutrophils by increasing their number, survival and recruitment to the site of infection [221]. Neutrophils are the transport vehicle for intracellular pathogens carrying antigens to DCs and participate in the activation of the T-cell immune response by DCs [222]. The reaction of neutrophils and macrophages during infection triggers the production of innate defence regulatory peptide 1 IDR1, with similar activity to defensins or cathelicidins [223]. IDR1 stimulates the antimicrobial activity of macrophages [224].

Along with activation of innate or adaptive immune cells, neutrophils, by releasing arginase or ROS, can inhibit NK-cell or T-cell activation by depriving extracellular levels of L-arginine, necessary for T-cell activation [225].

## 9. Conclusions

Superoxide is produced mainly through metabolic reactions in which oxygen molecules are consumed. The control of O_2_^•−^ represents for the cell the neural point in the balance between oxidants and antioxidants.

In a physiological equilibrium, reactive oxygen species (ROS) are useful substances involved in cell signalling. However, in a pathological state, ROS can be dangerous if the synthesis of these molecules is initiated in an uncontrolled manner. Consequently, they perform two tasks in the metabolism of the cell. The mitochondrial transport chain ETC generates superoxide under physiological conditions, increasing ROS production and oxidative stress. Mitochondria are the main source of intracellular O_2_^•−^ production, which can lead to mtDNA damage and increased superoxide production. Other internal sources that can induce superoxide are NADPH oxidase NOX, xanthine oxidase XO, lipoxygenase, cyclooxygenase, and cytochrome P450 CYP/cytochrome P450 reductase POR.

Reactive stress occurs when an excess of free radicals overwhelms the body and cannot be neutralized by the antioxidant mechanism (enzymes or peptides, such as glutathione or superoxide dismutase (SOD), etc.). At the same time, ROS-induced toxicity is beneficial for the oxidative destruction of microbial pathogens during the activation of the innate immune system via specialized immune cells, such as neutrophils or macrophages.

## Data Availability

Not applicable.
